# Mutations in the B.1.1.7 SARS-CoV-2 Spike Protein Reduce Receptor-Binding Affinity and Induce a Flexible Link to the Fusion Peptide

**DOI:** 10.3390/biomedicines9050525

**Published:** 2021-05-08

**Authors:** Eileen Socher, Marcus Conrad, Lukas Heger, Friedrich Paulsen, Heinrich Sticht, Friederike Zunke, Philipp Arnold

**Affiliations:** 1 Institute of Anatomy, Functional and Clinical Anatomy, Friedrich-Alexander-University Erlangen-Nürnberg (FAU), 91054 Erlangen, Germany; friedrich.paulsen@fau.de; 2 Institute for Clinical and Molecular Virology, Friedrich-Alexander-University Erlangen-Nürnberg (FAU), University Hospital Erlangen, 91054 Erlangen, Germany; 3 Division of Bioinformatics, Institute of Biochemistry, Friedrich-Alexander-University Erlangen-Nürnberg (FAU), 91054 Erlangen, Germany; mar.conrad@fau.de (M.C.); heinrich.sticht@fau.de (H.S.); 4 Laboratory of Dendritic Cell Biology, Department of Dermatology, Friedrich-Alexander-University Erlangen-Nürnberg (FAU), University Hospital Erlangen, 91052 Erlangen, Germany; lukas.heger@uk-erlangen.de; 5 Department of Operative Surgery and Topographic Anatomy, Sechenov University, 119992 Moscow, Russia; 6 Erlangen National High Performance Computing Center (NHR@FAU), Friedrich-Alexander-University Erlangen-Nürnberg (FAU), 91058 Erlangen, Germany; 7 Department of Molecular Neurology, Friedrich-Alexander-University Erlangen-Nürnberg (FAU), University Hospital Erlangen, 91054 Erlangen, Germany; friederike.zunke@fau.de

**Keywords:** COVID-19, SARS-CoV-2, B.1.1.7, molecular dynamics simulation, ACE2, receptor binding

## Abstract

The B.1.1.7 variant of the SARS-CoV-2 virus shows enhanced infectiousness over the wild type virus, leading to increasing patient numbers in affected areas. Amino acid exchanges within the SARS-CoV-2 spike protein variant of B.1.1.7 affect inter-monomeric contact sites within the trimer (A570D and D614G) as well as the ACE2-receptor interface region (N501Y), which comprises the receptor-binding domain (RBD) of the spike protein. However, the molecular consequences of mutations within B.1.1.7 on spike protein dynamics and stability or ACE2 binding are largely unknown. Here, molecular dynamics simulations comparing SARS-CoV-2 wild type with the B.1.1.7 variant revealed inter-trimeric contact rearrangements, altering the structural flexibility within the spike protein trimer. Furthermore, we found increased flexibility in direct spatial proximity of the fusion peptide due to salt bridge rearrangements induced by the D614G mutation in B.1.1.7. This study also implies a reduced binding affinity for B.1.1.7 with ACE2, as the N501Y mutation restructures the RBD–ACE2 interface, significantly decreasing the linear interaction energy between the RBD and ACE2. Our results demonstrate how mutations found within B.1.1.7 enlarge the flexibility around the fusion peptide and change the RBD–ACE2 interface. We anticipate our findings to be starting points for in depth biochemical and cell biological analyses of B.1.1.7.

## 1. Introduction

**Figure 1:**
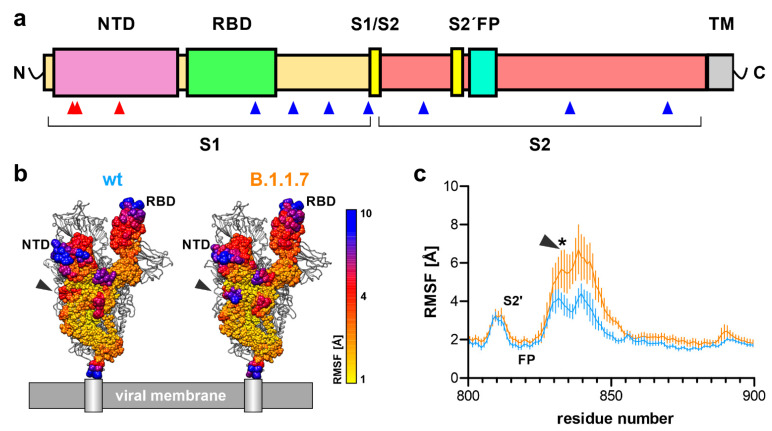
Structural flexibility in the B.1.1.7 SARS-CoV-2 S protein. (**a**) Schematic primary structure of the SARS-CoV-2 S protein indicating the site of amino acid deletions (red arrowheads) or single amino acid exchanges (blue arrowheads; NTD, N-terminal domain; RBD, receptor-binding domain; S1/S2, furin cleavage site at positions 685/686; S2′, furin/TMPRSS2 cleavage site at positions 815/816; FP, fusion peptide; TM, transmembrane domain and C-terminal end). (**b**) S protein trimer as it would reside on the cell surface with one subunit colored for structural flexibility as calculated during simulation (root-mean-square fluctuations (RMSF), *n* = 6). NTD denotes the N-terminal domain, RBD the receptor-binding domain and the grey arrowhead the loop region between amino acids 835-843. (**c**) Line plot of RMSF values for amino acid residues 800-900 reveals increased flexibility for residues 835 and 843 in B.1.1.7 (orange) when compared to wt (blue). The arrowhead denotes the same region as in (**b**), and the asterisk indicates statistical differences for these amino acids (*n* = 6; two-way ANOVA; statistical significance assumed for * *p* < 0.05; full statistic can be found in Table S2).

The outbreak of the severe acute respiratory syndrome (SARS) caused by the SARS-like coronavirus SARS-CoV-2 has become a global pandemic with daily increasing numbers of infections, and deaths exceeding 2.5 million world-wide [1]. On top of the rapid, global spread of SARS-CoV-2, new and more contagious virus variants comprise an additional threat [2,3,4,5]. The novel SARS-CoV-2 variant B.1.1.7 first emerged in southeast England in November 2020 and is estimated to be 56% more transmissible [6]. This variant is characterized by several amino acid deletions and exchanges, with most of the protein-coding mutations found within the surface-anchored spike (S) protein of the virus: del69–70HV, del144Y, N501Y, A570D, D614G, P681H, T761I, S982A, D1118H [7] (Figure S1a). The homotrimeric S protein facilitates viral entry into host cells by interaction of its receptor-binding domain (RBD) with the cell surface receptor angiotensin-converting enzyme 2 (ACE2) [8,9,10]. The S protein consist of a S1 subunit, harboring the receptor-binding domain, and a S2 subunit, which is essential for viral entry into the host cell after dissociation from S1 (Figure 1a, Figure S1b). Separation of the S1 and S2 subunits is mediated by host cell surface proteases at the S1/S2 and S2′ cleavage sites of the S protein [11,12]. Previous results suggest that dramatic structural changes of the S2 unit lead to the exposure of a fusion peptide that facilitates cell membrane fusion and virus entry [13]. For S protein priming, human serine protease TMPRSS2 cleavage within a multi-basic (furin) cleavage site between AAs685/686 (S1/S2 cleavage site) or AAs815/816 (S2′ cleavage site) has been proposed [9,14], and a recent study even suggests blocking SARS-CoV-2 infection by using a TMPRSS2 inhibitor [9]. Cellular uptake as well as the protein structure of the S protein of the SARS-CoV-2 virus have been studied extensively. Hence, cryo-electron microscopy and crystallographic analyses of the S protein trimer [15,16], ACE-2-bound S protein [17], as well as ACE-2-bound receptor-binding domain [18], have shed light onto the structural mechanisms of viral entry. Until now, this structural information has been missing for globally emerging SARS-CoV-2 variants, exhibiting several mutations within the S protein and higher infectivity. To date, we only have a vague understanding of the molecular reasons leading to enhanced contagion and cellular uptake of SARS-CoV-2 variants like the B.1.1.7 (British), but also the B.1.351 (South African) and P.1 (Brazilian/Japanese) variants. Interestingly, these three Variants of Concern (VOC) have the N501Y and D614G mutation in common [2,19]. 

In the present study, we focus on the B.1.1.7 variant and use molecular dynamics (MD) simulations of S protein trimer to assess its dynamic behavior in terms of conformational stability as well as interaction of the isolated viral RBD in complex with human ACE2 to calculate linear interaction energies for wild type and mutated (N501Y) RBD–ACE2 complexes. Our data suggest increased flexibility around the fusion peptide induced by the D614G mutation and reduced binding affinity between RBD and ACE2 due to conformational reorganization of the RBD–ACE2 interface mediated by N501Y. Upon proteolytic processing at the S2′ cleavage site (Arg815/Ser816), a conformational switch alters partial salt bridge formation between arginine 815 and negatively charged aspartate and glutamate residues in the vicinity to keep the new C-terminus in place via noncovalent interactions.

## 2. Materials and Methods

### 2.1. Generation of the Starting Structures

To generate starting structures for wild type, wild type with the D614G mutation (wt+D614G), and B.1.1.7 SARS-CoV-2, we used the protein sequence annotated in UniProt (www.uniprot.org; accessed on 22 December 2020) with the identifier P0DTC2 (SARS-CoV-2; wild-type) and changed the sequence in accordance with the reported deletions (del69-70, del144) for the SARS-CoV-2 B.1.1.7 variant. Using Swiss-Model Expasy (www.swissmodel.expasy.org), we generated a model for the SARS-CoV-2 wild type, wild type with the D614G mutation, and SARS-CoV-2 B.1.1.7 variant on the basis of the Cryo-EM solved structure of the triple ACE2 bound SARS-CoV-2 spike protein trimer (PDB ID: 7KMS) [17] and used chain A as a template. After modeling, structures were transferred into UCSF Chimera [20] and single amino acid exchanges were inserted into the B.1.1.7 structure using the *swapaa* command. These were N501Y, A570D, D614G, P681H, T716I, S982A and D1118H. Post-translational modifications (here sugars) were removed. Monomeric spike proteins of wt, wt+D614G and B.1.1.7 were then C3 symmetrized to generate trimeric spike protein complexes and used as starting structures for molecular dynamics (MD) simulations. Cleavage of wild type and the B.1.1.7 variant was structurally implemented in the PDB files used as starting structures by adding termination (TER) records (indicates end of a chain) between arginine 815 and serine 816 in all three chains of the trimer.

To investigate the interface between the RBD of the spike protein and ACE2, the respective wild type starting structure was obtained from the PDB database (PDB ID code: 7KMB) [17]. To also generate the starting structure for the MD simulations of the B.1.1.7 variant, the N501Y amino acid exchange was introduced with Swiss-PdbViewer 4.1.0.

### 2.2. Molecular Dynamics Simulations

Molecular dynamics simulations were performed using version 20 of the Amber Molecular Dynamics software package (ambermd.org) [21] and the ff14SB force field [22]. With the Amber Tool LEaP, all systems were electrically neutralized with Na^+^ ions and solvated with TIP3P [23] water molecules. The trimeric SARS CoV-2 spike protein was solvated in a cuboid water box with at least 15 Å distance from the borders to the solute, whereas the RBD complexed with ACE2 was solvated in a water box with the shape of a truncated octahedron and at least 25 Å distance from the borders to the solute.

The simulations followed a previously applied protocol [24]. At first, a minimization was carried out in three subsequent steps to optimize the geometry of the starting structures. In the first step of the minimization, the water molecules were minimized, while all remaining atoms were restrained with a constant force of 10 kcal·mol^−1^·Å^−2^ to the initial positions. In the second step, additional relaxation of the sodium ions and the hydrogen atoms of the protein were allowed, while the remaining protein was restrained with 10 kcal·mol^−1^·Å^−2^. In the last step, no restraints were used, so that the whole protein, the ions, and the water molecules were minimized. All three minimization parts started with 2500 steps using the steepest descent algorithm, followed by 2500 steps of a conjugate gradient minimization. After the minimization, the systems were equilibrated in two successive steps. In the first step, the temperature was raised from 10 to 310 K within 0.1 ns and the protein was restrained with a constant force of 5 kcal·mol^−1^·Å^−2^. In the second step (0.4 ns length), only the Cα atoms of the protein were restrained with a constant force of 5 kcal·mol^−1^·Å^−2^. In both equilibration steps, the time step was 2 fs. Minimization and equilibration were carried out on CPUs, whereas the subsequent production runs were performed using pmemd.CUDA on Nvidia A100 GPUs [7,25,26]. Subsequently, 200 ns (trimeric spike protein) or 500 ns (RBD complexed with ACE2) long production runs were conducted without any restraints and at 310 K (regulated by a Berendsen thermostat [27]). Furthermore, the constant pressure periodic boundary conditions were used with an average pressure of 1 bar and isotropic position scaling. For bonds involving hydrogen, the SHAKE algorithm [28] was applied in the equilibration and production phase. To accelerate the production phase of the molecular dynamics (MD) simulations, hydrogen mass repartitioning (HMR [29] was used in combination with a time step of 4 fs. The MD simulations were performed two times for all forms of the trimeric spike protein and four times for both forms of the RBD in complex with ACE2.

Trajectory analysis (analysis of root-mean-square deviation of atomic positions (RMSD), root-mean-square fluctuations (RMSF), analysis of native contacts, measurement of interatomic distances, calculation of linear interaction energy (electrostatic and van der Waals interactions, used default cutoff of 12.0 Å) was carried out using the Amber tool cpptraj [30]. Contacts were evaluated with an in-house Perl script parsing the trajectory using the prior named Amber tools and assigning contacts based on a distance criterion of ≤5 Å between any pair of atoms, as was performed previously [31].

### 2.3. Statistics and Display

Statistical analyses were generated with GraphPad Prism (v.8.0.0 for Windows, GraphPad Software, San Diego, California USA, www.graphpad.com) and statistical tests were applied as indicated below the figure. Plots were generated in GraphPad and Gnuplot (v.4.6). All structure images were made with UCSF Chimera 1.15 [20].

## 3. Results

### 3.1. Flexibility of the Spike Trimer

To compare flexibility between the SARS-CoV-2 wild type (wt) and B.1.1.7 variant, root-mean-square fluctuation (RMSF) values were calculated for the backbone atoms of all individual residues over 200 ns simulation time. Thereafter, RMSF values were averaged for all six calculated monomers (two runs with three subunits per trimer) and visualized as spheres of different diameter and color (Figure 1b, Figure S2a,b). Highest RMSF values were calculated for the RBD with flexibility of up to 10 Å, especially at the interface positions where interaction with ACE2 would occur upon receptor binding (Figure S2a–c). In the N-terminal domain (NTD), deletions of amino acids 69, 70 and 144 induce a reduced flexibility for amino acid residues arginine 78, leucine 249 and threonine 250 in the B.1.1.7 variant (Figure S2d, Table S1). These residues are all located at the surface of the spike protein and are most exposed to interact with other spike trimers at the viral surface (Figure S2b,e). The loop region C-terminal of the S2′ cleavage site and the fusion peptide showed increased flexibility in the B.1.1.7 variant when compared to wt (Figure 1c, Figure S2c). Flexibility increased from a maximum of 4 Å in wt to a maximum of about 7 Å in B.1.1.7. Residues 835-843 showed markedly increased RMSF values (Table S2). To understand this change in flexibility, we analyzed the salt bridges formed by charged residues of this amino acid stretch.

### 3.2. D614G Induces Flexibility around the Fusion Peptide via Salt Bridge Rearrangement

**Figure 2:**
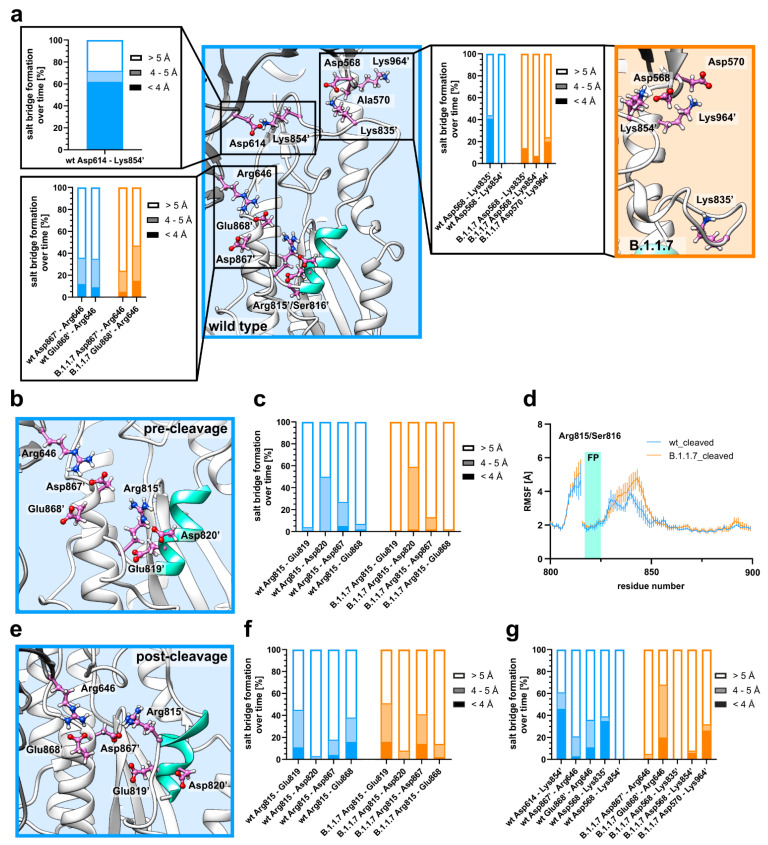
Salt bridge stability is altered upon D614 mutation in pre- and postcleavage state. (**a**) Stability of a fusion peptide (aquamarine) adjacent loop region is mediated by salt bridge formation between aspartate 614 (Asp614) from one chain and lysine 854 (Lys854′; ‘denotes residues from a neighboring chain) from the neighboring chain in wild type (wt; blue background). An additional salt bridge is formed by aspartate 568 (Asp568) and lysine 835 (Lys835′) from the neighboring chain in wt. In B.1.1.7 (orange background) this interaction is weaker and the newly inserted aspartate at position 570 (A570D) forms an additional salt bridge with lysine 964 (Lys964′). In close proximity to arginine 815 (part of the S2′ cleavage site), ionic interaction was measured between arginine 646 (Arg646) and aspartate 867 (Asp867′) and glutamate 868 (Glu868′). (**b**) Structural representation of the wt precleavage state (continuous polypeptide chain; similar in B.1.1.7) with negatively charged residues glutamate 819 (Glu819), aspartate 820 (Asp820), aspartate 867 (Asp867) and glutamate 868 (Glu868) shown around arginine 815 (Arg815). (**c**) Percentage of salt bridge formation over time for four negatively charged residues with arginine 815 in the precleavage state. (**d**) Average RMSF values plotted against the residue numbers for wt and B.1.1.7 after in silico proteolytic cleavage at the S2′ site (aquamarine bar; FP = fusion peptide). (**e**) Structural representation of the wt postcleavage state (discontinuous polypeptide chain with a break between arginine 815 and serine 816; similar in B.1.1.7) with negatively charged residues glutamate 819 (Glu819), aspartate 820 (Asp820), aspartate 867 (Asp867) and glutamate 868 (Glu868) shown around arginine 815 (Arg815). (**f**) Percentage of salt bridge formation over time for four different residue pairs in the postcleavage state. (**g**) Percentage of salt bridge formation over time for the stabilizing salt bridge pairs as analyzed in (**a**).

We found lysine 835 and lysine 854 as positively charged amino acid residues in the region of this increased flexibility. Analyzing the wild type structure during MD simulation revealed interchain salt bridge formation between lysine 835 from one chain and aspartate 568 from the neighboring chain and interchain salt bridge formation between lysine 854 and aspartate 614 (Figure 2a). We also identified interchain salt bridges between arginine 646 and glutamate 868 and aspartate 867 (Figure 2a), although to a lesser extent than the other two described charged residue pairs. To analyze the consistency of these identified salt bridges, we measured the interatomic distances over all three trimer subunits for both simulation runs. Therefore, we defined distances between the carbon atoms of the carboxyl groups of aspartate or glutamate and the nitrogen of the NH_3_^+^ group in the side chain of lysine or the carbon atom in ζ-position (most distal carbon in the side chain) in arginine. We separated the results into under 4 Å (preferred distance for salt bridge formation), between 4 and 5 Å (important for arginine residues due to the usage of the carbon atom in ζ-position) and above 5 Å (for no salt bridge formation). The interchain salt bridge, which can only be present in wild type, formed by aspartate 614 and lysine 854, is at a preferred distance of below 4 Å in about 60% of the simulation time. This indicates a certain importance for this salt bridge for local stability. For the salt bridge formed by lysine 835 from one subunit and aspartate 568 from an adjacent subunit we found distances smaller 4 Å in over 40% of the time for the wt S protein (Figure 2a). Additionally, there was no interaction found between lysine 854 and aspartate 568, arguing for a stable conformation of this fusion peptide adjacent loop region in the wild type S protein, as it is supported by two salt bridges. Analyzing the same salt bridges in the B.1.1.7 variant reveals a different salt bridge pattern. As aspartate 614 is missing (D614G), lysine 854 is unmatched in this variant. We found that both lysine residues in this region, lysine 854 and lysine 835 form partial salt bridges with aspartate 568. However, even combined, both residues are only in the preferred distance of under 4 Å for about 20% of the time. To analyze the direct effect of the D614G mutation in this matter, we also calculated MD simulations of a wt variant with an inserted glycine for aspartate at position 614 (wt+D614G). Calculating the RMSF values also revealed an increase in flexibility in the region of lysine 854 (Figure S3a). Analyzing the distances between lysine residues 854 and 835 with aspartate 568 also revealed a partial interaction between lysine 854 and aspartate 568 as found in the B.1.1.7 variant (Figure S3b). However, the interaction between lysine 835 and aspartate 568 is as strong as in wt, indicating an additional destabilizing mechanism in the B.1.1.7 variant. This additional destabilization in B.1.1.7 could be explained by the A570D mutation (Figure 2a, orange box). The newly inserted aspartate engages with lysine 964 in about 20% of the time in a preferred salt bridge distance of under 4 Å and could thereby destabilize interactions of aspartate 568 (Figure 2a). For all three variants, wt, wt+D614G and B.1.1.7, partial interaction between arginine 646 and aspartate 867 or glutamate 868 was found (Figure 2a, Figure S3b). As these residues are in close proximity of arginine 815, which is part of the S2′ cleavage site, we also analyzed interactions of this residue with surrounding negatively charged residues (Figure 2b) and found them with aspartate 820 and, to a lesser extent, with aspartate 867 in this precleavage state (Figure 2c). To analyze the fate of arginine 815 upon cleavage by, e.g., TMPRSS2, we performed in silico cleavage between arginine 815 and serine 816 and simulated wt and B.1.1.7. The RMSF values showed the known pattern of increased flexibility for B.1.1.7 (Figure 2d), and we also analyzed salt bridges (Figure 2e). In contrast to the precleavage state, where arginine 815 is in contact with aspartate 820, this interaction is lost in the postcleavage state and arginine 815 engages with glutamate 819 in wt and B.1.1.7. In wt, an additional salt bridge forms in the postcleavage state between arginine 815 and glutamate 868, while the interaction with aspartate 867 remains similar (Figure 2f). In contrast, arginine 815 from B.1.1.7 engages mainly with aspartate 867 in the postcleavage state, and there is only a small interaction with glutamate 868 (Figure 2f). These salt bridge interactions indicate a noncovalent stabilization after proteolytic priming at the S2′ position that requires a certain conformational rearrangement in this area. We also reanalyzed the stabilizing salt bridges between aspartate 614 and lysine 854 and aspartate 568 and lysine 835 in wt (Figure 2g) and found almost no change compared to precleavage conditions (Figure 2a). For B.1.1.7, we found that the small interaction between aspartate 568 and lysine 835 was completely lost in the postcleavage state and only the small interaction between aspartate 568 and lysine 854 remains in B.1.1.7 (Figure 2g). Of note, we also identified that interactions between arginine 646 and aspartate 867 and glutamate 868 are different in wt and B.1.1.7. While there is a more even distribution of contact formation between arginine 646 and the two negatively charged residues in wt, there is a clear preference towards glutamate 868 in B.1.1.7 (Figure 2g). Taken together, we identified how the D614 mutation (in combination with the A570D mutation) induces a loss of conformational stability that increases flexibility in a pre- and postcleavage state. We also identified changes in salt bridge engagement of arginine 815 between a precleavage (continuous protein chain) and postcleavage state (discontinuous protein chain), that then stabilizes the new C-terminus via salt bridge rearrangement. To analyze whether this increase in flexibility in B.1.1.7 is also propagated towards the RBD over the entire S protein or is a local phenomenon, we analyzed the RMSD values over the simulation time for the RBDs (Figure S3c–e). The RMSD values were low and very similar for the variants analyzed (wt, wt+D614G and B.1.1.7). Thus, we hypothesized that changes in RBD behavior are a direct effect of the N501Y mutation and further analyzed it.

### 3.3. N501Y Replacement Displaces Glutamine 498 from the SARS-CoV-2 ACE2 Interface

To analyze binding of the S protein to the ACE2 receptor, we used the isolated RBD in complex with ACE2 (PDB ID code: 7KMB [17]) as starting structure for molecular dynamics simulations. The only amino acid exchange localized in the RBD is N501Y, which resides at the RBD–ACE2 interface (Figure S1a). Simulation for 500 ns revealed no marked differences in structural flexibility between wt and B.1.1.7, as it can be deduced from the RMSF values (Figure S4a,b). Compared to the MD simulation of the trimeric S protein without ACE2 (unbound state), the overall flexibility was very similar, but the bound RBD (RBD–ACE2 complex) showed markedly lower RMSF values in the region between RBD residue 475 and residue 520, where most of the RBD residues are located which closely interact with ACE2 (Figure S4c). To identify changes between wt and B.1.1.7, we analyzed contacts below 5 Å over time. For the B.1.1.7 variant we identified that glutamine 498 loses almost all contacts to ACE2 residues aspartate 38 and lysine 353, while the number of contacts is strongly reduced for tyrosine 41 (Figure S5a, Table S3). We also identified a markedly increased number of contacts for the newly inserted tyrosine at position 501 to lysine 353 in B.1.1.7 when compared to the wt asparagine residue (Figure S5a, individual runs shown in Figures S7 and S8). Structural analysis revealed the expulsion of glutamine 498 from the RBD–ACE2 interface due to the bulkier tyrosine side chain in the B.1.1.7 variant (Figure S5b,c). We also noticed a change in side chain conformation for lysine 353, explaining the altered contact numbers (Figure S5b,c). We were then interested in individual binding energies and decomposed the RBD–ACE2 interface.

### 3.4. The N501Y Mutation Decreses Electrostatic Binding in B.1.1.7

**Figure 3:**
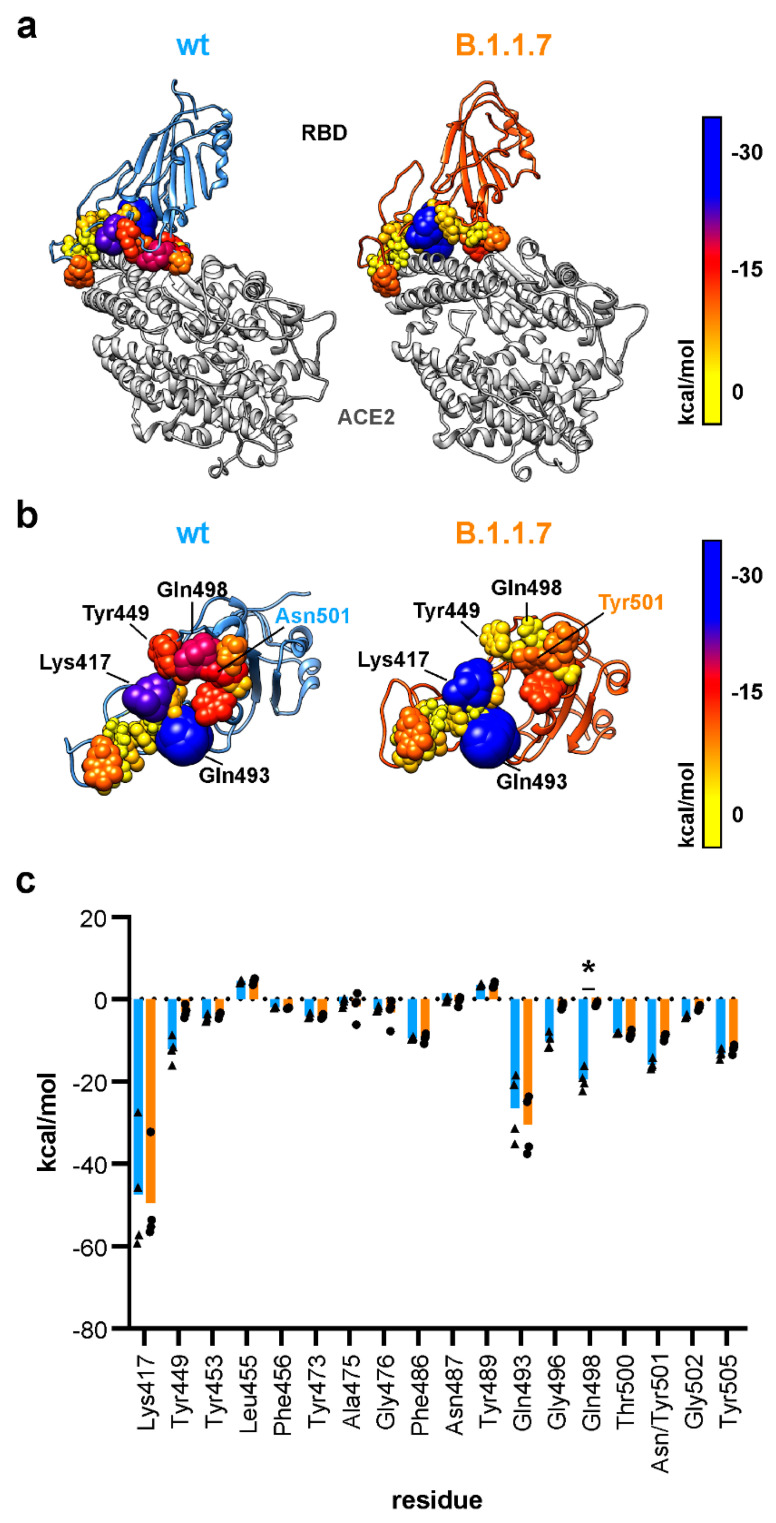
Electrostatic affinity between the RBD and ACE2. (**a**) Structural representation of the RBD (wild type, wt: blue; B.1.1.7: orange) in complex with ACE2 (grey). RBD residues with atoms within a maximum distance of 4 Å from ACE2 are shown as spheres with radii and colors according to their electrostatic linear interaction energy to ACE2. (**b**) View of the interacting interface of the RBD with ACE2. Residue color and atom size represent their electrostatic linear interaction energy to ACE2. (**c**) Quantification of electrostatic linear interaction energy for all residues within 4 Å distance of ACE2 (*n* = 4; two-way ANOVA; statistical significance assumed for * *p* < 0.05; full list of results in Table S4).

To decompose the electrostatic linear interaction energy, all residues within 4 Å of ACE2 were analyzed. We identified the main electrostatic interaction in the central part of the RBD–ACE2 interface for wt and B.1.1.7. The electrostatic interaction is comprised by interaction of glutamine 493, lysine 417 and glutamine 498 with ACE2 in the wt RBD (Figure 3a,b). Regarding the B.1.1.7 variant, glutamine 493 and lysine 417 also contribute greatly to electrostatic interaction between RBD and ACE2. However, glutamine 498, which is displaced from the binding interface, does not contribute to electrostatic interaction to the same degree as in wt (Figure 3a,b).Consequently, markedly reduced electrostatic interaction was calculated for B.1.1.7 (Figure 3c, Table S4). Decomposition of electrostatic interaction on the individual residue level for ACE2 reveals that lysine 353 loses its interaction with the RBD in the B.1.1.7 variant (Figure S9a,b, Table S5). This fits with our data on contact formation, as lysine 353 from ACE2 and glutamine 498 from the RBD lose all their contacts in the B.1.1.7 variant (Figure S5a). In total, we calculated an electrostatic interaction energy of -200 kcal/mol for wt and -165 kcal/mol in B.1.1.7. Computationally more demanding calculations of the binding energy using Molecular Mechanics Generalized Born Surface Area (MM/GBSA) also imply a reduced binding affinity (Figure S6). As tyrosine is a bulkier residue than asparagine at position 501, we were then interested in van der Waals contacts also at the level of the individual residues.

### 3.5. Tyrosine at Position 501 Increases Van der Waals Interaction Locally in B.1.1.7

**Figure 4:**
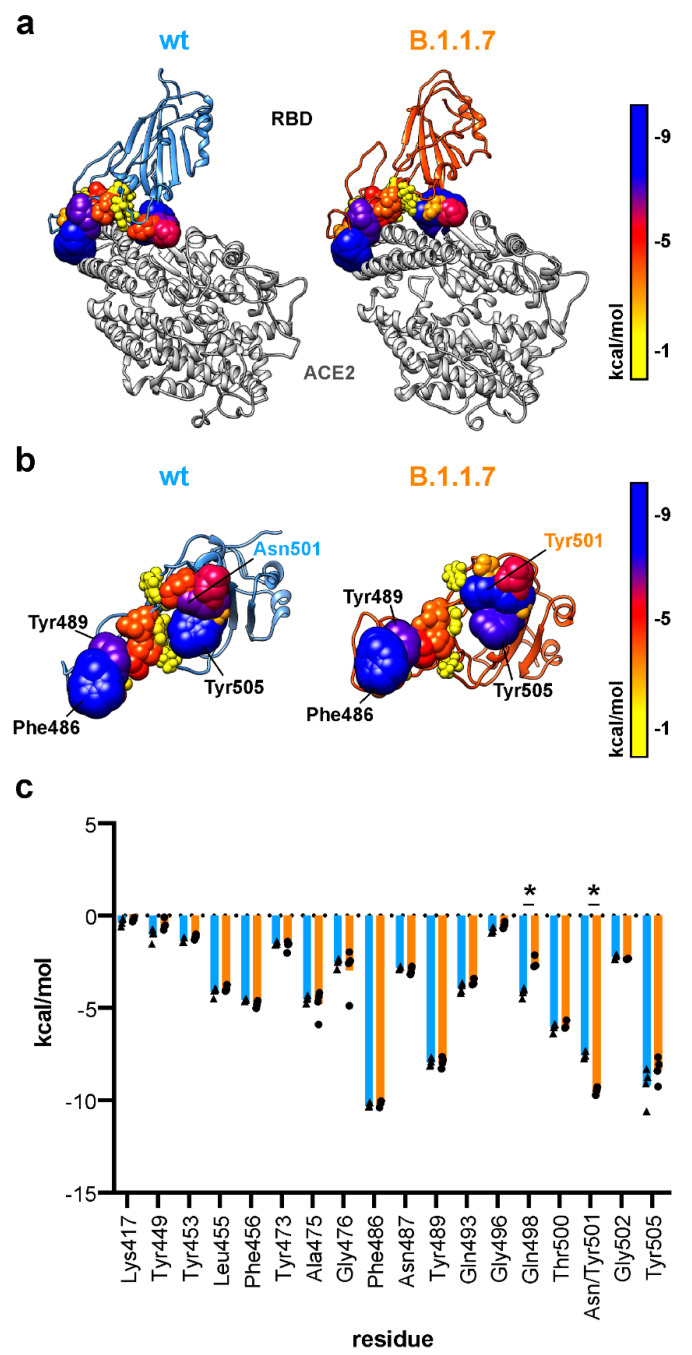
Van der Waals linear interaction energy between the RBD and ACE2. (**a**) Structural representation of the RBD (wild type, wt: blue; B.1.1.7: orange) in complex with ACE2. RBD residues with atoms within a maximum distance of 4 Å from ACE2 are shown as spheres with radii and colors according to their van der Waals linear interaction energy to ACE2. (**b**) View of the interacting interface of the RBD with ACE2. Residue color and atom size represent the van der Waals linear interaction energy to ACE2. (**c**) Quantification of van der Waals linear interaction energy for all RBD residues within 4 Å distance of ACE2 (*n* = 4; two-way ANOVA; statistical significance assumed for * *p* < 0.05; full list of results in Table S6).

Decomposition of individual van der Waals linear interaction energies identifies the edges of the RBD–ACE2 interface as important regions. Van der Waals interactions are mainly facilitated by phenylalanine 486, tyrosine 489 and tyrosine 505 in both wt and B.1.1.7 (Figure 4a,b). A major difference was calculated for mutated residue 501 accounting for an asparagine in wt and tyrosine in B.1.1.7 (Figure 4c, Table S6). Here we found an increased van der Waals interaction energy in B.1.1.7. Van der Waals interaction energies did not differ in total and were calculated to be around −90 kcal/mol for both wt and B.1.1.7. The van der Waals linear interaction energy was lowered for tyrosine 41 and glutamine 42 from ACE2 (Figure S10a,b, Table S7).

## 4. Discussion

We used MD simulations on (i) S protein trimers and (ii) S protein RBD–ACE2 complexes to compare wt and B.1.1.7 variants. Understanding the dynamic stability of the S protein is of vital importance to comprehend molecular mechanisms underlying viral entry into the host cell. In particular, this could help to assess the superior infectivity of newly emerging viral variants. Although MD simulation data on wt S protein RBDACE2 interaction has been reported [32,33], data on dynamic changes induced by the N501Y variant found in B.1.1.7 (British), B.1.351 (South African) and P.1 (Brazilian, Japanese) is lacking [7]. These three variants also harbor the D614G mutation [7], which has previously been associated with higher infectivity [5,34,35,36] and viral replication [34,37]. For variants carrying this D614G mutation, it has also been suggested that more functional S protein is incorporated into the virion membrane [5]. However, molecular mechanisms explaining the higher infectivity of the D614G mutation are still elusive. Our data, derived from simulations of the entire S trimer and comparing wt with B.1.1.7, reveal a rearrangement of salt bridges caused by the loss of interaction between position 614 due to the D614G mutation and lysine 854 in B.1.1.7. This induced flexibility at residues C-terminal of the fusion peptide is enhanced in postcleavage conformations, as salt bridges formed by aspartate 568 are also largely lost in B.1.1.7. Release of the fusion peptide might allow a faster insertion of the latter into the host cell membrane due to the increased flexibility of the adjacent linker. This might also happen after cleavage at the S2′ position by TMPRSS2 [9,38] or allow faster entry into host cells after priming the S2′ position by furin during protein secretion [9], especially as enhanced proteolytic cleavage was proposed for D614G S proteins [39]. MD simulation also reveals structural stability of the postcleavage state induced by salt bridge formation involving arginine 815, which would allow such priming during protein secretion without destabilizing the overall structural integrity. The newly formed salt bridge of aspartate 570 with lysine 964 does not compensate for the loss of aspartate 614 in terms of protein stability, but might weaken salt bridges formed by aspartate 568. Additionally, as aspartate 614 comes from the S1 region and lysine 854 from the S2 region of the S protein, dissociation of the S1 and S2 domain of the S protein, important for membrane fusion after entry of the fusion peptide into the host cell membrane, might be enhanced [1]. Furthermore, we show that deletions in the NTD reduce flexibility in the outer region of the S trimer, potentially allowing more spike proteins at the virion host cell interface of B.1.1.7 and thus increasing the probability of viral uptake [5].

Binding to ACE2 via the RBD of the viral S protein is the first step of cellular uptake and infection. Previous reports have already highlighted the importance of residues lysine 417, tyrosine 449, glutamine 493 and glutamine 498 on the RBD of the S protein for interface formation with ACE2 [10,33]. On ACE2, residues aspartate 30, lysine 31 and lysine 353 were also identified as important interactors with the RBD of the S protein [10,33,40]. We were also able to confirm the importance of the above-mentioned residues in interface formation between RBD and ACE2 here. In addition, our data suggest that the insertion of the bulkier tyrosine at position 501, as found in B.1.1.7, B.1.351 and P.1, excludes glutamine 498 from the RBD–ACE2 interface and thereby alters the molecular architecture of the RBD–ACE2 interface of the S protein. We measured a reduced electrostatic affinity of glutamine 498 from the S protein to residues on ACE2. For tyrosine 501 from the S protein RBD, we found an increased van der Waals interaction with lysine 353 (ACE2) in the N501Y variant. Similar RMSD values of the RBD for wt and B.1.1.7 suggest that amino acid deletions/exchanges in the NTD and fusion peptide adjacent loop region cause local changes and do not change the overall flexibility of the RBD.

In conclusion, our data suggest a higher mobility of the fusion peptide after release and reduced electrostatic affinity of the S protein RBD to ACE2 in B.1.1.7. This combination could allow faster membrane fusion of the virion with the host cell membrane and could be explained by (i) faster insertion of the fusion peptide and (ii) faster dissociation of the S protein RBD–ACE2 complex due to reduced electrostatic affinity. Dissociation of the S protein components is important for efficient membrane fusion after insertion of the fusion peptide. Our data might serve as a basis for advanced biochemical and cell biological experiments to better understand the importance of individual amino acid exchanges for viral pathology.

## Supplementary Materials

The following are available online at https://www.mdpi.com/article/10.3390/biomedicines9050525/s1, Figure S1: Structural representation of the SARS-CoV-2 S protein on the viral surface, Figure S2: Differences of RMSF values between wt and B.1.1.7 spike protein residues, Figure S3: Results of the wt+D614G variant and RMSD values the receptor-binding domain (RBD), Figure S4: Structural flexibility of the receptor-binding domain-ACE2 (RBD–ACE2) complex, Figure S5: Analysis of the interface between ACE2 and the receptor-binding domain (RBD), Figure S6: Interaction energies of the MM/GBSA analyses for the wild type and the B.1.1.7 receptor-binding domain (RBD) variants complexed with ACE2, Figure S7: Individual intermolecular contact plots for all four molecular dynamics (MD) simulation runs of the wild type RBD–ACE2 complex, Figure S8: Individual intermolecular contact plots for all four molecular dynamics (MD) simulation runs of the B.1.1.7 RBD–ACE2 complex, Figure S9: Electrostatic affinity shown on the RBD–ACE2 interface for ACE2 residues, Figure S10: Van der Waals linear interaction energy shown on the RBD–ACE2 interface for ACE2 residues, Table S1: Statistical analysis of RMSF plots from wt and B.1.1.7 spike protein residues 26-300, Table S2: Statistical analysis of RMSF plots from wt and B.1.1.7 spike protein residues 800-900, Table S3: Statistical analysis of contacts between residues from the receptor binding domain and ACE2 from wt and B.1.1.7, Table S4: Statistical analysis of electrostatic interaction energy of residues from the receptor binding domain comparing wt and B.1.1.7, Table S5: Statistical analysis of electrostatic interaction energy of residues from ACE2 comparing wt and B.1.1.7, Table S6: Statistical analysis of van der Waals interaction energy of residues from the receptor binding domain comparing wt and B.1.1.7, Table S7: Statistical analysis of electrostatic interaction energy of residues from ACE2 comparing wt and B.1.1.7.
