# Supplementary material for: Mutations in the B.1.1.7 SARS-CoV-2 Spike Protein Reduce Receptor-Binding Affinity and Induce a Flexible Link to the Fusion Peptide
Source: Biomedicines. 2021 May 8;9(5):525. doi: 10.3390/biomedicines9050525 (PMC8151884; doi:10.3390/biomedicines9050525)
Supplement: Supplementary file 1 [file biomedicines-09-00525-s001.zip › biomedicines-1211717-SI.pdf]

Mutations in the B.1.1.7 SARS-CoV-2 spike protein reduce receptor-binding affinity and induce a flexible link to the fusion peptide

Eileen Socher, Marcus Conrad, Lukas Heger, Friedrich Paulsen, Heinrich Sticht,

Friederike Zunke and Philipp Arnold

Supplementary Materials

Supplementary Figures

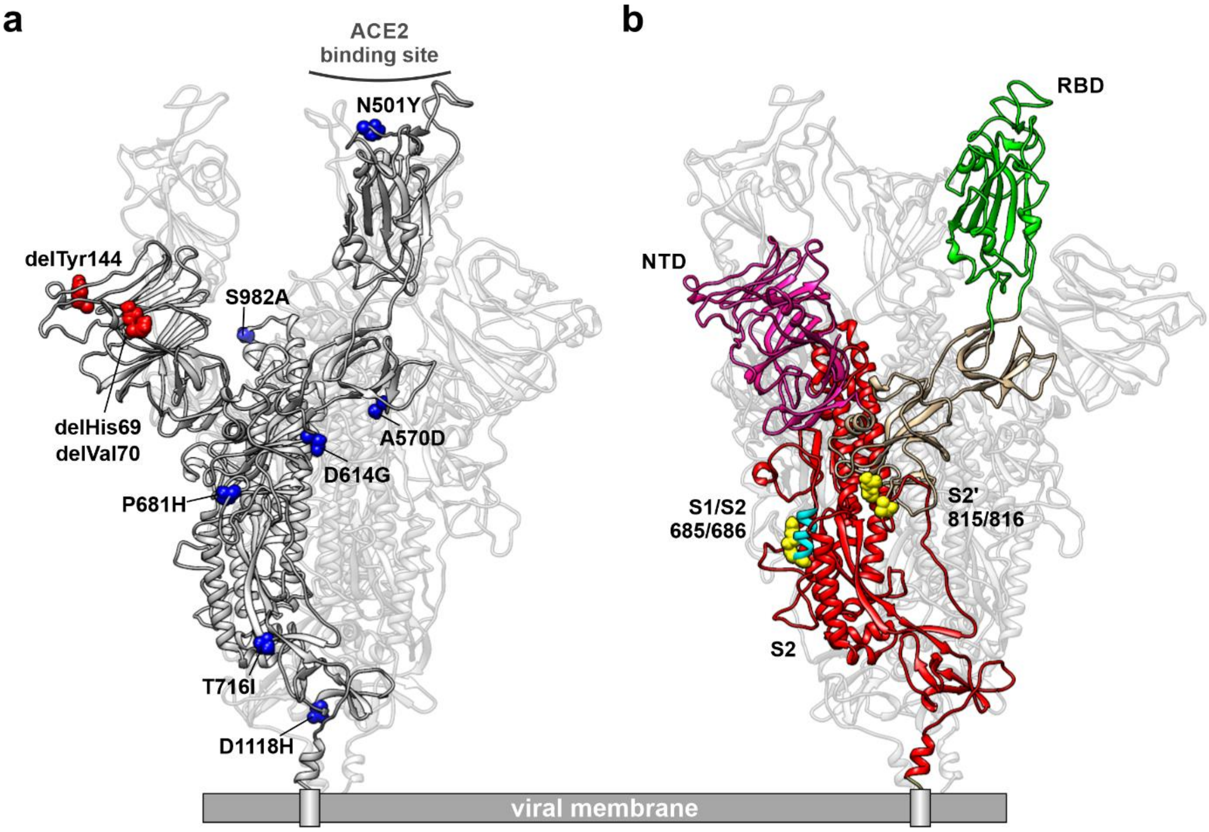

Figure S1. Structural representation of the SARS-CoV-2 S protein on the viral surface (**a**) with amino acid residues shown as spheres that are deleted (red) or mutated (blue) in the B.1.1.7 variant and (**b**) with domains highlighted in color as in Figure 1a (N-terminal domain, NTD in pink; receptor-binding domain, RBD in green; S1 region in khaki; S1/S2 and S2′ cleavage sites in yellow with amino acids as spheres; fusion peptide in cyan; S2 domain in red).

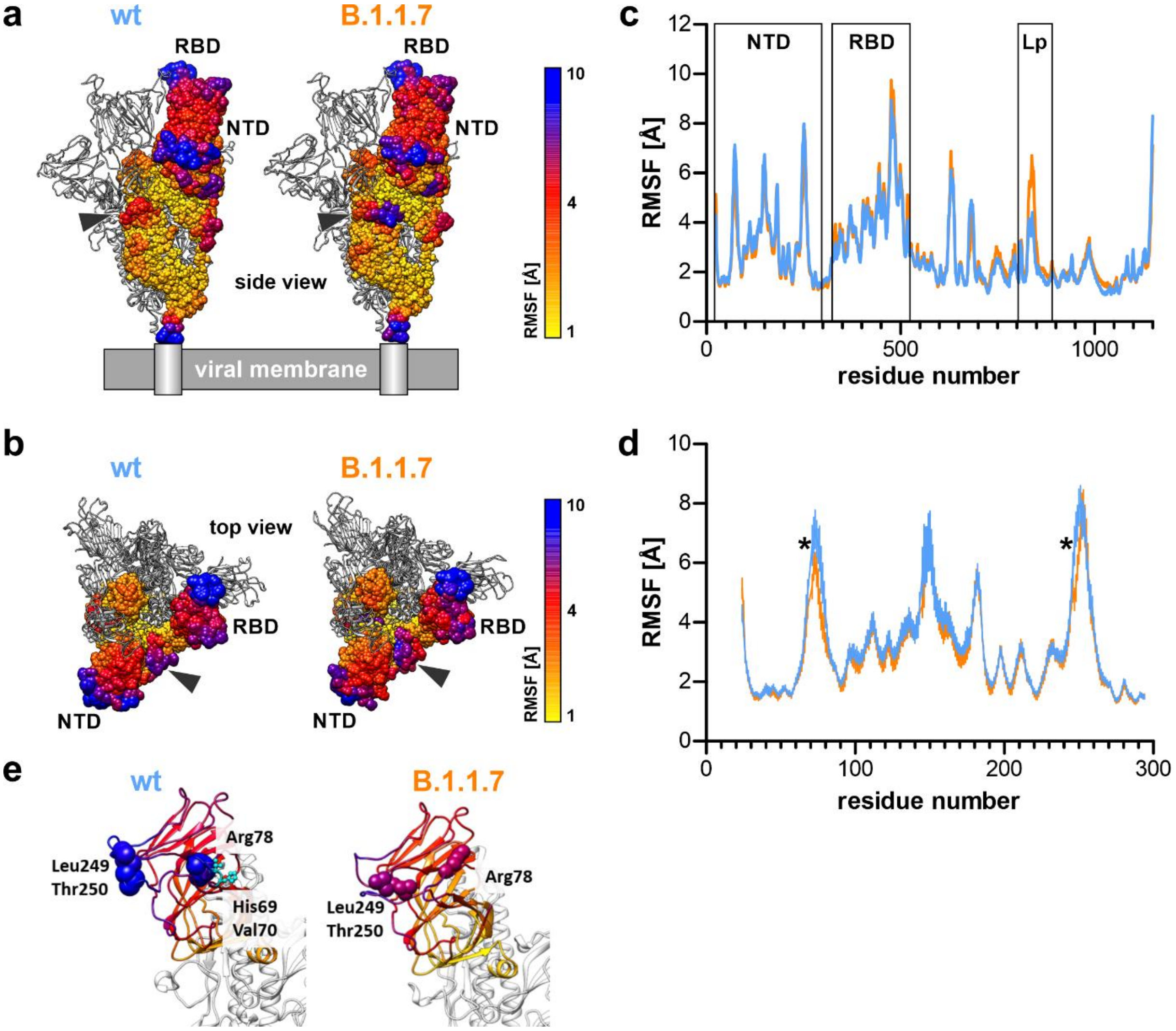

Figure S2. Differences of RMSF values between wt and B.1.1.7 spike protein residues. (**a**) Structural representation of the SARS-CoV-2 S protein in side view on the viral surface with residues shown as colored spheres for one subunit according to their RMSF value (grey arrowhead, flexible loop region in B.1.1.7). (**b**) Top view of (a). (**c**) Line plot of RMSF values for calculated averages of six subunits of the wt (blue) and B.1.1.7 (orange) S protein. (**d**) Line plot of RMSF values averaged over six subunits of the N-terminal domain from wt (blue) and B.1.1.7 (orange). Asterisks indicate positions of significant differences (n = 6; two-way ANOVA; significance assumed for $p<0.05$; full list in Table S1). (**e**) Structural representation of the N-terminal domain colored according to RMSF values. Residues that differ significantly in RMSF values are represented as spheres. Additionally, His69 and Val70 are shown for wild type (wt) and are missing as del69,70 in B.1.1.7 (n = 6; two-way ANOVA; significance assumed for *$p<0.05$; full list in Table S1). NTD = N-terminal domain, RBD = receptor-binding domain, Lp = loop region around amino acids 836-844.

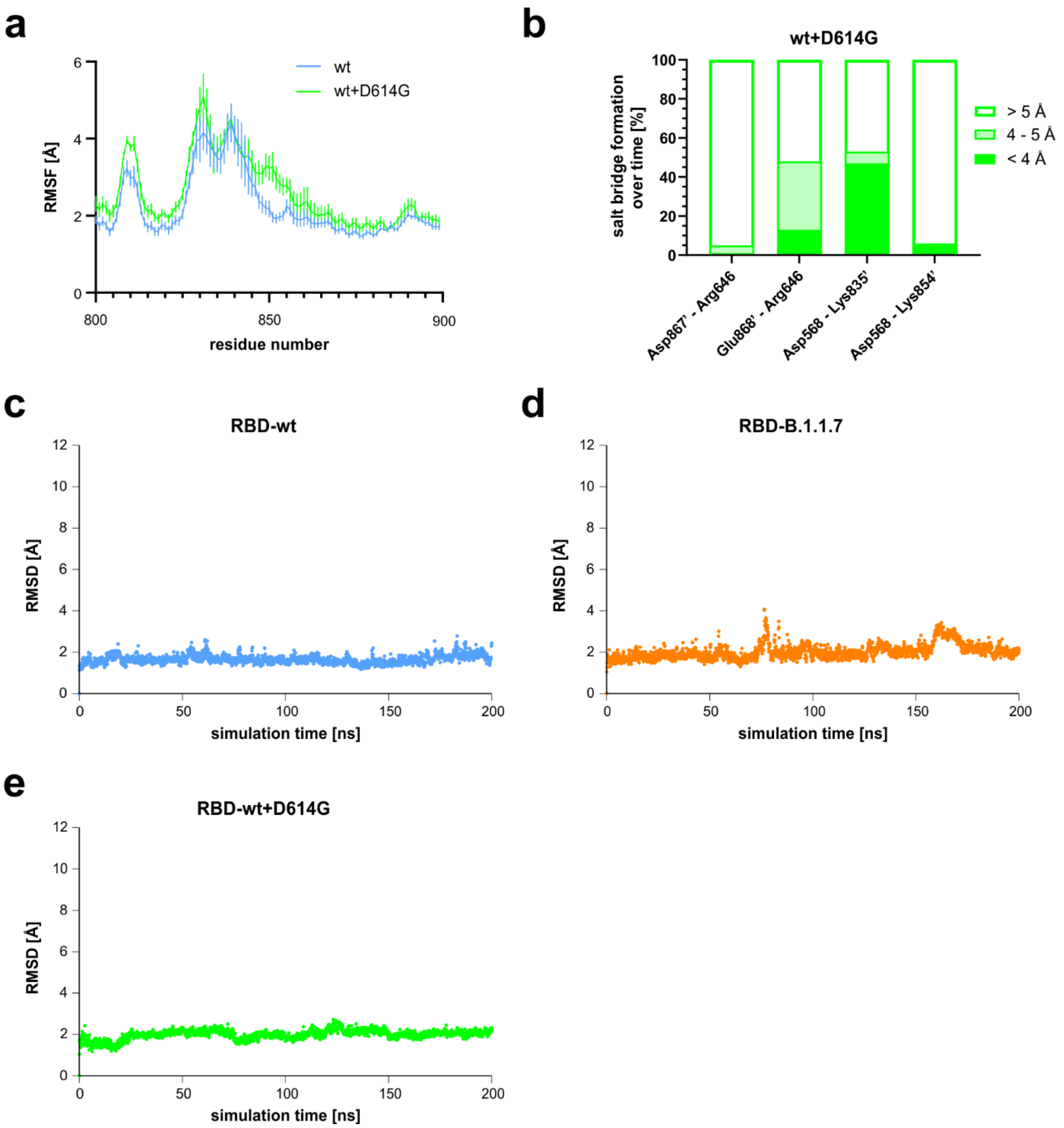

Figure S3. Results of the wt+D614G variant and RMSD values the receptor-binding domain (RBD). (**a**) Line plot of root-mean-square fluctuation (RMSF) values for amino acid residues 800 to 900. Averages were calculated for all three trimer subunits and both simulation runs of the wild type (wt, blue) and wt+D614G variant (green). (**b**) Percentage of salt bridge formation over time for four different residue pairs. All residue pairs represent interchain interactions with residues from two different, but directly neighboring chains within the trimeric spike protein. (**c**-**e**) Conformational stability was measured as root-mean-square deviation (RMSD) values over simulation time for the receptor-binding domain (RBD) of (c) the wild type (d) the B.1.1.7 variant and (e) the wild type with the D614G mutation. One representative plot is shown for wild type spike protein and its variants.

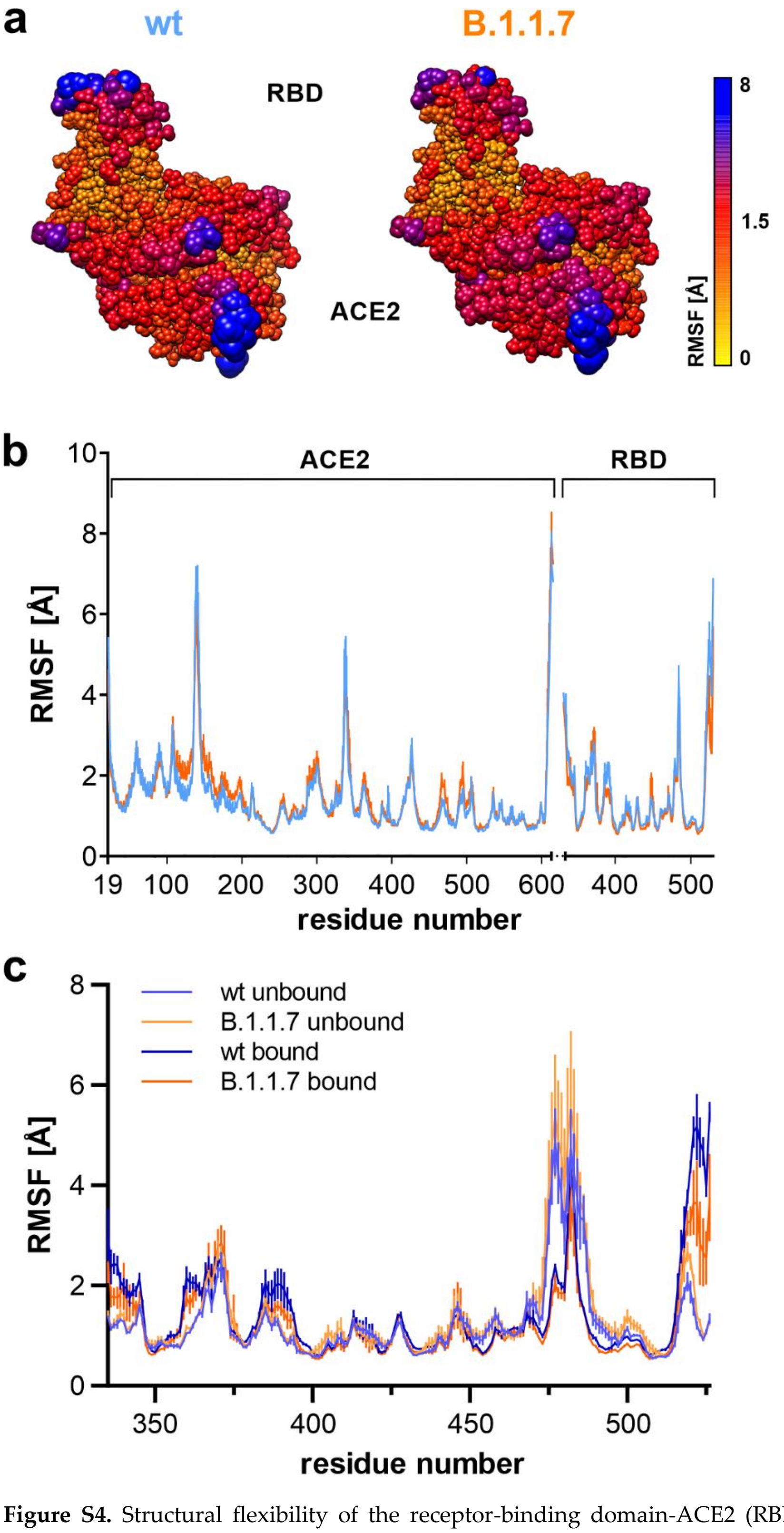

Figure S4. Structural flexibility of the receptor-binding domain-ACE2 (RBD-ACE2) complex. (**a**) Residues are colored and shown as spheres of different size according to their individual RMSF values. RBD denotes the receptor-binding domain and ACE2 the angiotensin-converting enzyme 2. (**b**) Line plot of RMSF values averaged over four molecular dynamics (MD) simulation runs for the RBD-ACE2 complex with wild type (wt) in blue and the B.1.1.7 variant in orange. (**c**) Line plot of RMSF values from the RBD in the unbound state (from the S protein trimer MD simulations) and in the bound state to ACE2 (from the RBD-ACE2 complex MD simulations).

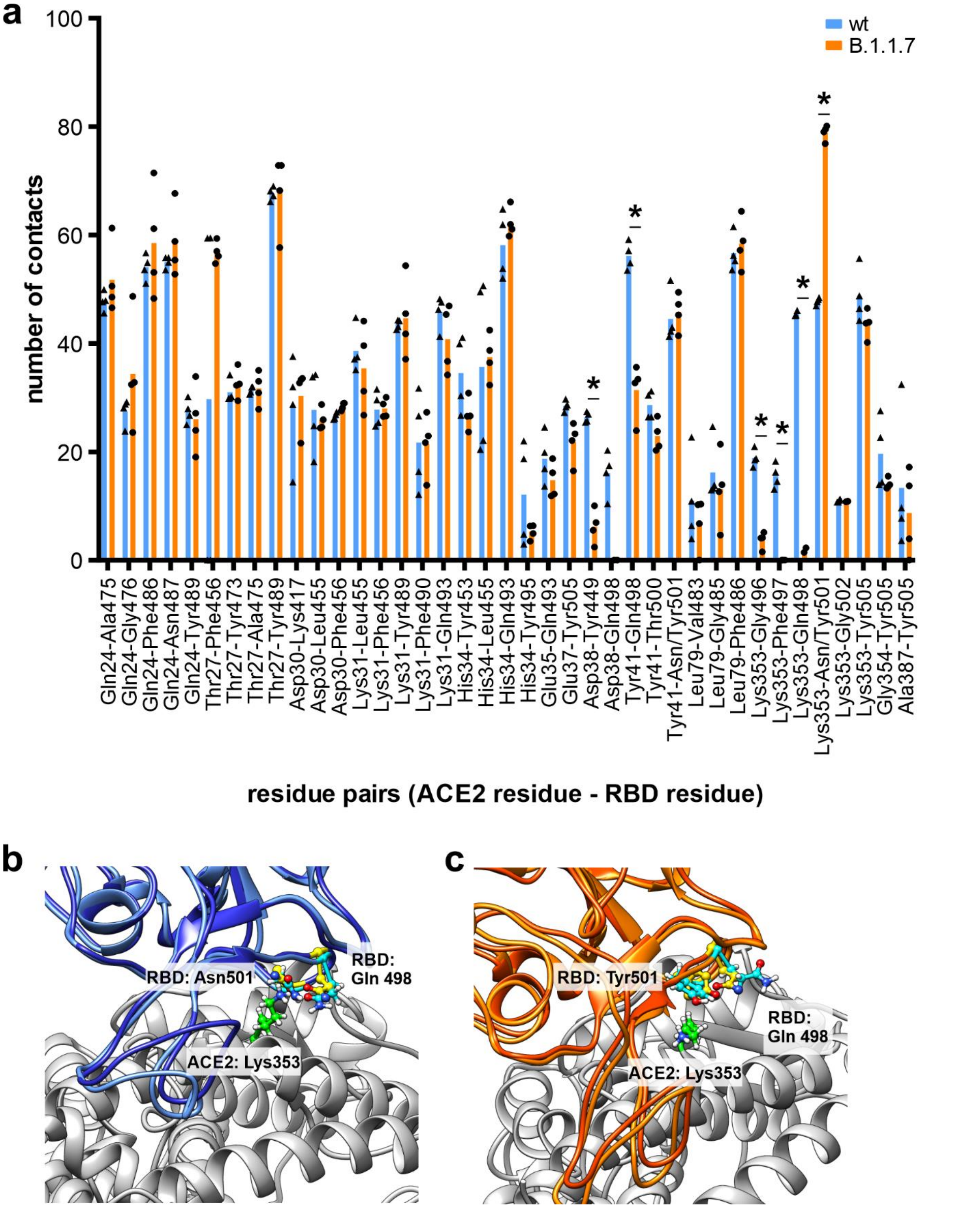

Figure S5. Analysis of the interface between ACE2 and the receptor-binding domain (RBD). (**a**) Average number of contacts for residues from ACE2 and the RBD. Residues were included when they came in closer proximity than 5 Å. Loss of contacts were calculated for glutamine 498 (Gln498) in B.1.1.7, and gain of contacts for the newly inserted tyrosine at position 501 (Tyr501) with lysine 353 (Lys353). Wild type (wt) is shown in blue and B.1.1.7 in orange (n = 4, two-way ANOVA; statistical significance was assumed for *$p<0.05$; full list of results in Table S3). (**b**,**c**) Structure of the RBD-ACE2 interface with residues asparagine 501 (Asn501; wild type in blue) or tyrosine 501 (Tyr501; B.1.1.7 in orange), glutamine 498 (Gln498) (all from RBD) and lysine 353 (green) from ACE2 shown in ball-and-stick. Yellow residues represent the starting conformation and residues in cyan are representative residue side chain conformations acquired during molecular dynamics simulation.

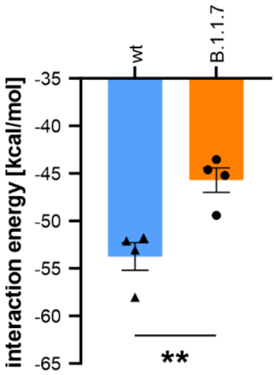

Figure S6. Interaction energies of the MM/GBSA analyses for the wild type and the B.1.1.7 receptor-binding domain (RBD) variants complexed with ACE2 ($n = 4$; two-sided Student's T-test; significance assumed for ** = $p < 0.01$).

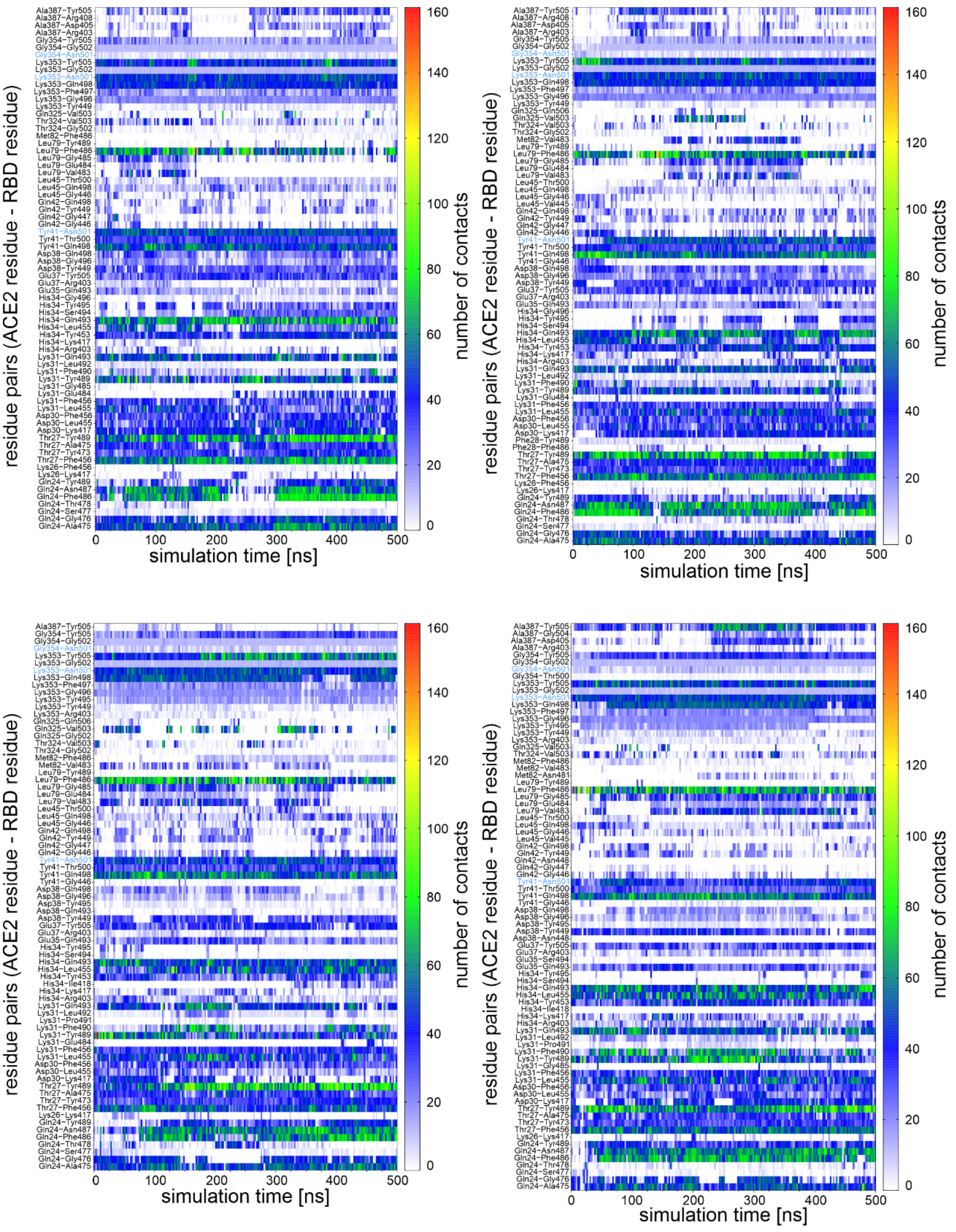

Figure S7. Individual intermolecular contact plots for all four molecular dynamics (MD) simulation runs of the wild type RBD-ACE2 complex. The number of contacts, calculated as the number of interresidue atom pairs that are within a maximum distance of 5 Å from each other, was plotted color-coded for intermolecular residue pairs over the simulation time. For instance, the residue pair Lys353-Asn501, with Lys353 expressed on ACE2 and Asn501 expressed on the RBD, has in all four MD simulations a calculated number of contacts between 40 and 60 over the whole simulation time. Residue pairs with asparagine 501 are highlighted in blue.

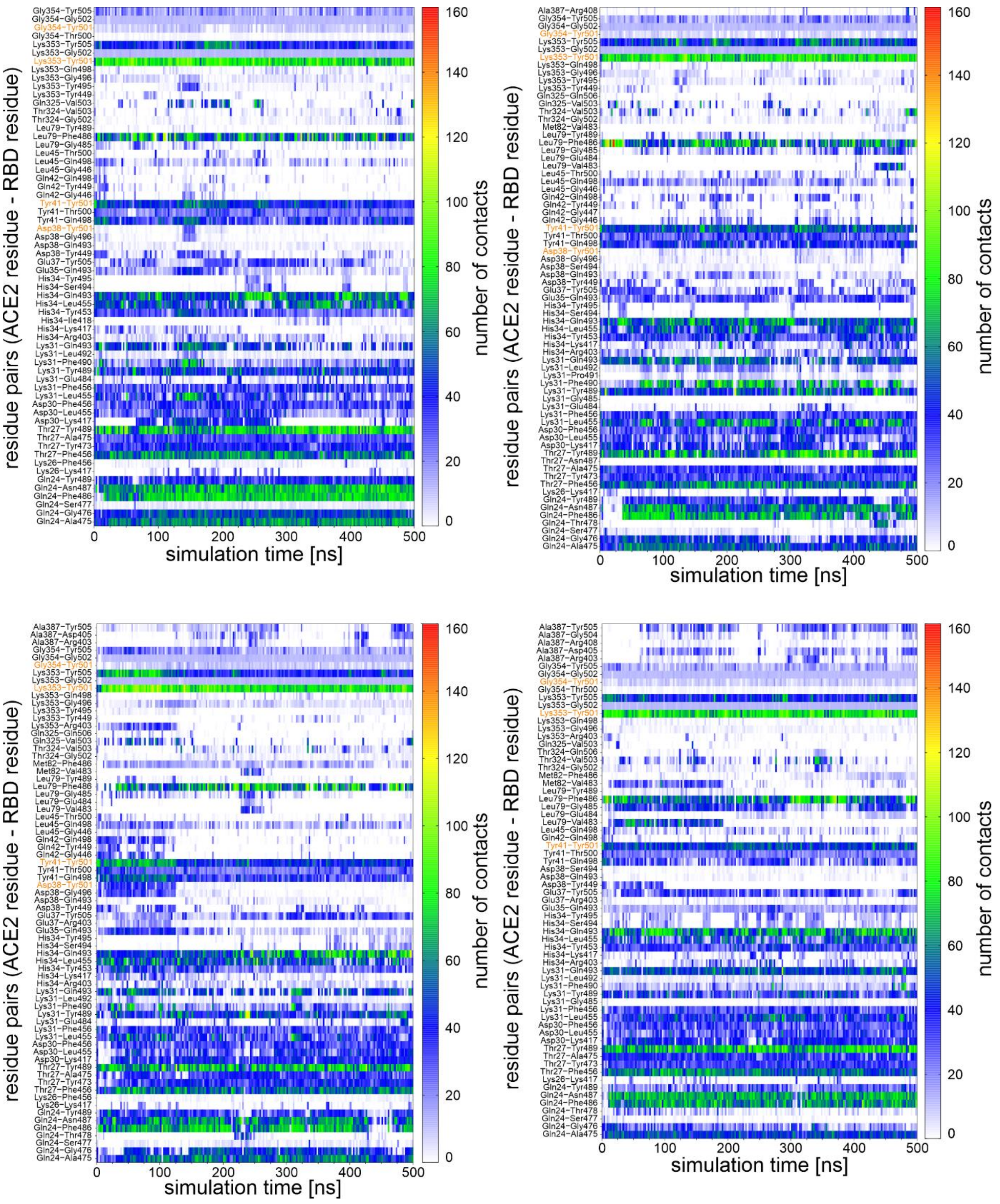

Figure S8. Individual intermolecular contact plots for all four molecular dynamics (MD) simulation runs of the B.1.1.7 RBD-ACE2 complex. The number of contacts, calculated as the number of interresidue atom pairs that are within a maximum distance of 5 Å from each other, was plotted color-coded for intermolecular residue pairs over the simulation time. For instance, the residue pair Lys353-Tyr501, with Lys353 expressed on ACE2 and Tyr501 expressed on the mutated RBD, has in all four MD simulations a calculated number of around 80 contacts over the whole simulation time. Residue pairs with mutated tyrosine 501 are highlighted in orange.

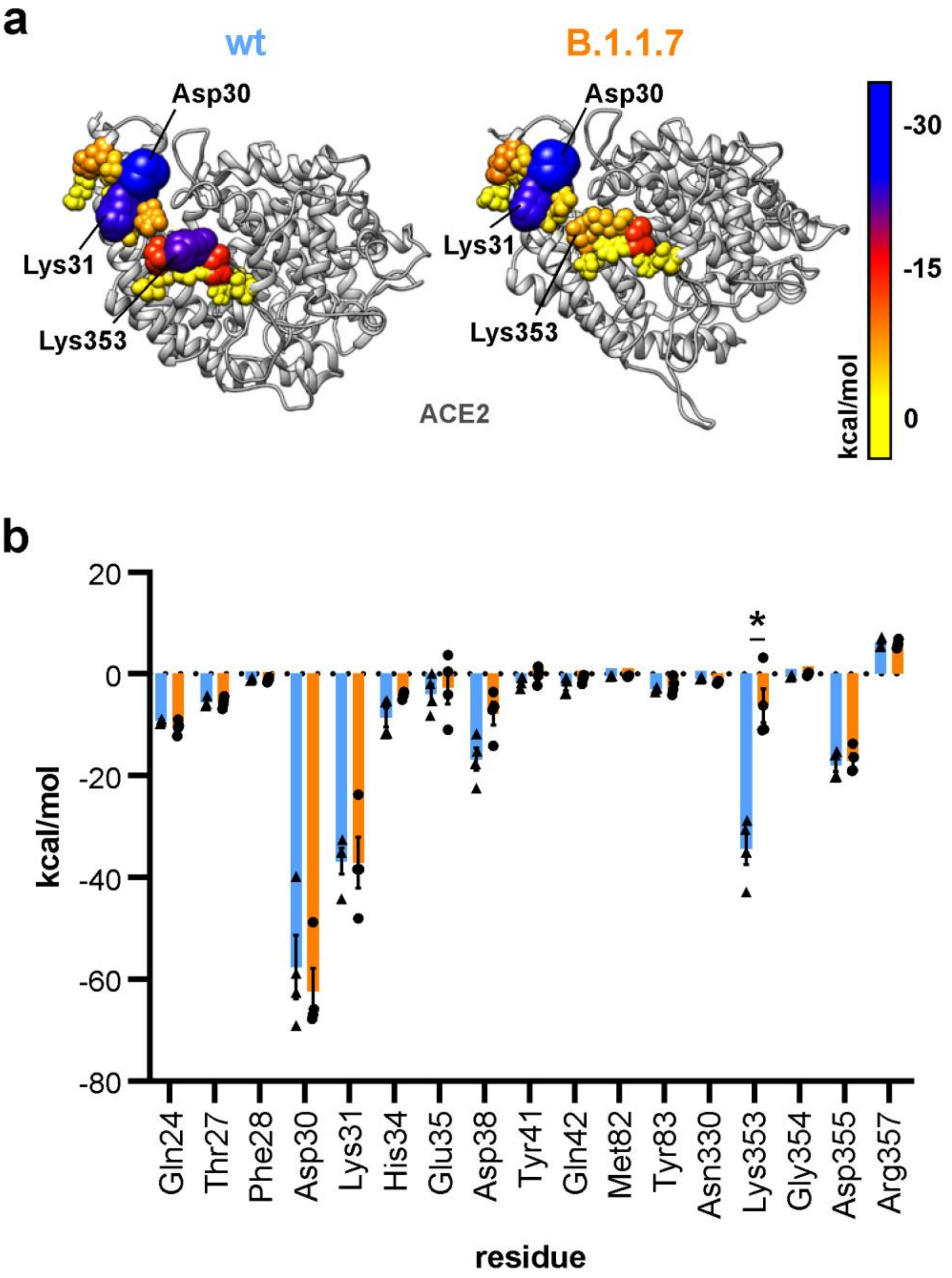

Figure S9. Electrostatic affinity shown on the RBD-ACE2 interface for ACE2 residues. (**a**) Residues in closer contact than 4 Å are shown as spheres of different size and color according to their electrostatic affinity with the RBD. (**b**) Quantification of the electrostatic linear interaction energy for all residues within a radius of 4 Å around the receptor-binding domain (n = 4; two-way ANOVA; statistical significance was assumed for *p<0.05; full list of results in Table S5).

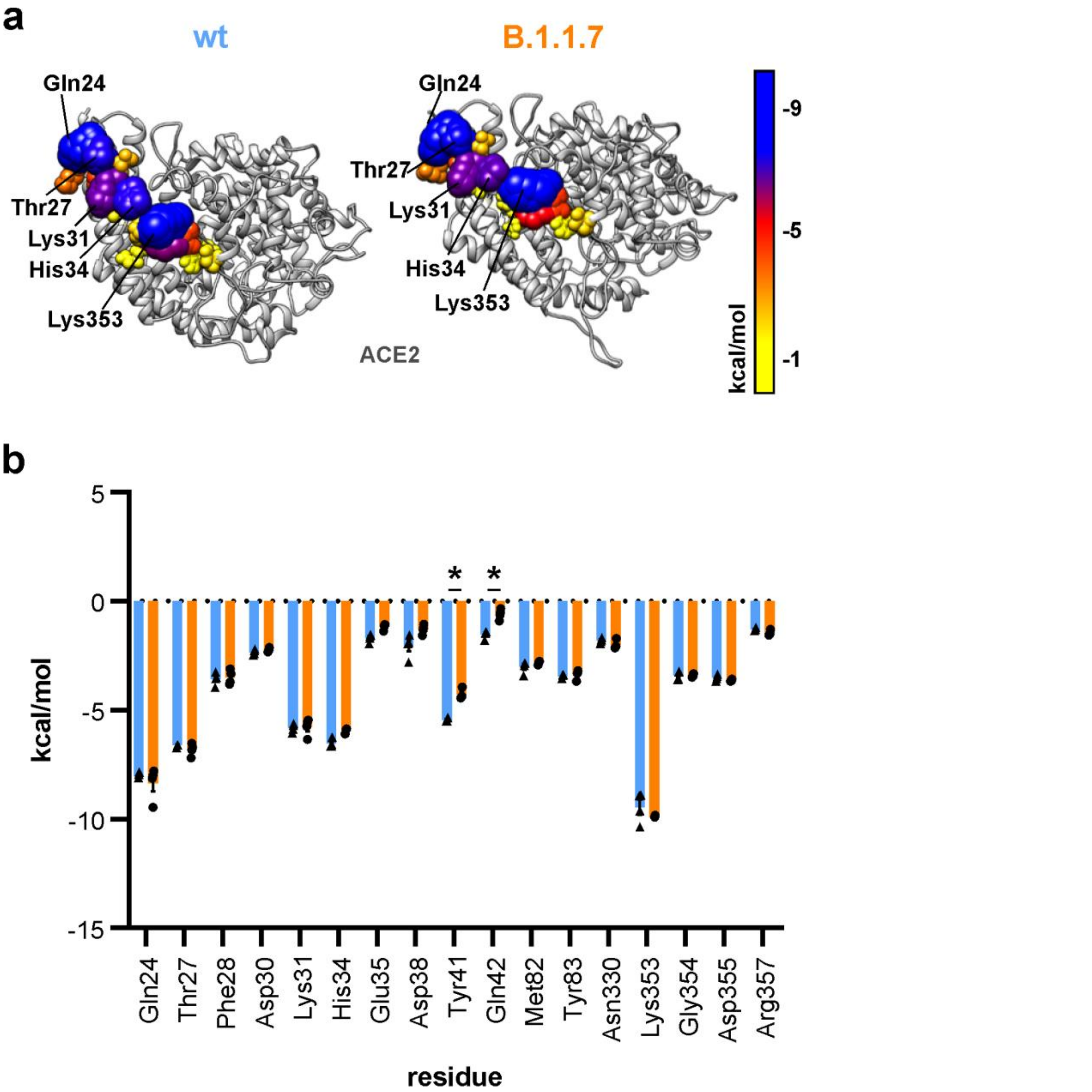

Figure S10. Van der Waals linear interaction energy shown on the RBD-ACE2 interface for ACE2 residues. (**a**) Residues in closer contact than 4 Å are shown as spheres of different size and color according to their van der Waals linear interaction energy with the RBD. (**b**) Quantification of the van der Waals linear interaction energy for all residues within a radius of 4 Å around the receptor-binding domain (n = 4; two-way ANOVA; statistical significance was assumed for *p<0.05; full list of results in Table S7).

Supplementary Tables

Table S1. Statistical analysis of RMSF plots from wt and B.1.1.7 spike protein residues 26-300 (n =6; two-way ANOVA; significance assumed for *p<0.05).

Compare each cell mean with the other cell mean in that row					
Number of families	1				
Number of comparisons per family	268				
Alpha	0.05				
Sidak's multiple comparisons test	Mean Diff,	95,00% CI of diff,	Significant?	Summary	Adjusted P Value
residue number					
26	-0.8329	-2.402 to 0.7364	No	ns	>0.9999
27	-0.6585	-2.228 to 0.9108	No	ns	>0.9999
28	0.008633	-1.561 to 1.578	No	ns	>0.9999
29	0.07843	-1.491 to 1.648	No	ns	>0.9999
30	0.1215	-1.448 to 1.691	No	ns	>0.9999
31	0.1201	-1.449 to 1.689	No	ns	>0.9999
32	0.09778	-1.472 to 1.667	No	ns	>0.9999
33	0.1022	-1.467 to 1.671	No	ns	>0.9999
34	0.09672	-1.473 to 1.666	No	ns	>0.9999
35	0.1199	-1.449 to 1.689	No	ns	>0.9999
36	0.1115	-1.458 to 1.681	No	ns	>0.9999
37	0.1039	-1.465 to 1.673	No	ns	>0.9999
38	0.05178	-1.518 to 1.621	No	ns	>0.9999
39	0.01918	-1.550 to 1.588	No	ns	>0.9999
40	0.01925	-1.550 to 1.589	No	ns	>0.9999
41	0.01545	-1.554 to 1.585	No	ns	>0.9999
42	-0.02265	-1.592 to 1.547	No	ns	>0.9999
43	0.001033	-1.568 to 1.570	No	ns	>0.9999
44	0.02818	-1.541 to 1.597	No	ns	>0.9999
45	0.07722	-1.492 to 1.647	No	ns	>0.9999
46	0.1215	-1.448 to 1.691	No	ns	>0.9999
47	0.1766	-1.393 to 1.746	No	ns	>0.9999
48	0.1580	-1.411 to 1.727	No	ns	>0.9999
49	0.1641	-1.405 to 1.733	No	ns	>0.9999
50	0.1397	-1.430 to 1.709	No	ns	>0.9999
51	0.1227	-1.447 to 1.692	No	ns	>0.9999
52	0.1026	-1.467 to 1.672	No	ns	>0.9999
53	0.08602	-1.483 to 1.655	No	ns	>0.9999
54	0.04920	-1.520 to 1.619	No	ns	>0.9999
55	0.05165	-1.518 to 1.621	No	ns	>0.9999
56	0.04797	-1.521 to 1.617	No	ns	>0.9999
57	0.06490	-1.504 to 1.634	No	ns	>0.9999
58	0.06837	-1.501 to 1.638	No	ns	>0.9999
59	0.05853	-1.511 to 1.628	No	ns	>0.9999
60	0.03623	-1.533 to 1.606	No	ns	>0.9999
61	0.05272	-1.517 to 1.622	No	ns	>0.9999
62	0.05685	-1.512 to 1.626	No	ns	>0.9999
63	0.1154	-1.454 to 1.685	No	ns	>0.9999
64	0.1579	-1.411 to 1.727	No	ns	>0.9999
65	0.1976	-1.372 to 1.767	No	ns	>0.9999
66	0.2034	-1.366 to 1.773	No	ns	>0.9999
67	0.3064	-1.263 to 1.876	No	ns	>0.9999
68	0.5059	-1.063 to 2.075	No	ns	>0.9999
71	1.499	-0.07048 to 3.068	No	ns	0.0935
72	1.663	0.09340 to 3.232	No	ns	0.207
73	1.384	-0.1854 to 2.953	No	ns	0.2346
74	1.084	-0.4849 to 2.654	No	ns	0.9295

75	1.143	-0.4268 to 2.712	No	ns	0.8280
76	1.106	-0.4634 to 2.675	No	ns	0.8981
77	1.355	-0.2140 to 2.925	No	ns	0.2878
78	1.853	0.2833 to 3.422	Yes	**	0.0029
79	1.558	-0.01083 to 3.128	No	ns	0.0552
80	1.443	-0.1268 to 3.012	No	ns	0.1493
81	1.040	-0.5289 to 2.610	No	ns	0.9721
82	0.9768	-0.5925 to 2.546	No	ns	0.9956
83	0.5730	-0.9963 to 2.142	No	ns	>0.9999
84	0.2178	-1.352 to 1.787	No	ns	>0.9999
85	0.09842	-1.471 to 1.668	No	ns	>0.9999
86	0.07445	-1.495 to 1.644	No	ns	>0.9999
87	0.07285	-1.496 to 1.642	No	ns	>0.9999
88	0.04062	-1.529 to 1.610	No	ns	>0.9999
89	0.04402	-1.525 to 1.613	No	ns	>0.9999
90	0.06403	-1.505 to 1.633	No	ns	>0.9999
91	0.1190	-1.450 to 1.688	No	ns	>0.9999
92	0.1667	-1.403 to 1.736	No	ns	>0.9999
93	0.2036	-1.366 to 1.773	No	ns	>0.9999
94	0.2328	-1.337 to 1.802	No	ns	>0.9999
95	0.2515	-1.318 to 1.821	No	ns	>0.9999
96	0.2559	-1.313 to 1.825	No	ns	>0.9999
97	0.09010	-1.479 to 1.659	No	ns	>0.9999
98	-0.04817	-1.617 to 1.521	No	ns	>0.9999
99	-0.04390	-1.613 to 1.525	No	ns	>0.9999
100	0.2482	-1.321 to 1.818	No	ns	>0.9999
101	0.2960	-1.273 to 1.865	No	ns	>0.9999
102	0.2943	-1.275 to 1.864	No	ns	>0.9999
103	0.2991	-1.270 to 1.868	No	ns	>0.9999
104	0.2596	-1.310 to 1.829	No	ns	>0.9999
105	0.2179	-1.351 to 1.787	No	ns	>0.9999
106	0.1703	-1.399 to 1.740	No	ns	>0.9999
107	0.1356	-1.434 to 1.705	No	ns	>0.9999
108	0.1081	-1.461 to 1.677	No	ns	>0.9999
109	0.09800	-1.471 to 1.667	No	ns	>0.9999
110	0.08167	-1.488 to 1.651	No	ns	>0.9999
111	0.1226	-1.447 to 1.692	No	ns	>0.9999
112	0.1433	-1.426 to 1.713	No	ns	>0.9999
113	0.1239	-1.445 to 1.693	No	ns	>0.9999
114	0.1017	-1.468 to 1.671	No	ns	>0.9999
115	0.09857	-1.471 to 1.668	No	ns	>0.9999
116	0.1169	-1.452 to 1.686	No	ns	>0.9999
117	0.1326	-1.437 to 1.702	No	ns	>0.9999
118	0.1560	-1.413 to 1.725	No	ns	>0.9999
119	0.1954	-1.374 to 1.765	No	ns	>0.9999
120	0.2262	-1.343 to 1.795	No	ns	>0.9999
121	0.2807	-1.289 to 1.850	No	ns	>0.9999
122	0.2631	-1.306 to 1.832	No	ns	>0.9999
123	0.2266	-1.343 to 1.796	No	ns	>0.9999
124	0.2003	-1.369 to 1.770	No	ns	>0.9999
125	0.3811	-1.188 to 1.950	No	ns	>0.9999
126	0.3791	-1.190 to 1.948	No	ns	>0.9999
127	0.3244	-1.245 to 1.894	No	ns	>0.9999
128	0.2923	-1.277 to 1.862	No	ns	>0.9999
129	0.2453	-1.324 to 1.815	No	ns	>0.9999
130	0.2129	-1.356 to 1.782	No	ns	>0.9999
131	0.1849	-1.384 to 1.754	No	ns	>0.9999
132	0.1594	-1.410 to 1.729	No	ns	>0.9999
133	0.1386	-1.431 to 1.708	No	ns	>0.9999
134	0.1484	-1.421 to 1.718	No	ns	>0.9999
135	0.1306	-1.439 to 1.700	No	ns	>0.9999
136	0.03127	-1.538 to 1.601	No	ns	>0.9999

137	0.1496	-1.420 to 1.719	No	ns	>0.9999
138	0.2176	-1.352 to 1.787	No	ns	>0.9999
139	0.2701	-1.299 to 1.839	No	ns	>0.9999
140	0.1339	-1.435 to 1.703	No	ns	>0.9999
141	0.09455	-1.475 to 1.664	No	ns	>0.9999
142	-0.02252	-1.592 to 1.547	No	ns	>0.9999
143	-0.1161	-1.685 to 1.453	No	ns	>0.9999
145	0.5314	-1.038 to 2.101	No	ns	>0.9999
146	0.8515	-0.7178 to 2.421	No	ns	>0.9999
147	1.087	-0.4820 to 2.657	No	ns	0.9257
148	1.281	-0.2884 to 2.850	No	ns	0.4617
149	0.4073	-1.162 to 1.977	No	ns	>0.9999
150	0.2902	-1.279 to 1.860	No	ns	>0.9999
151	0.2379	-1.331 to 1.807	No	ns	>0.9999
152	0.1059	-1.463 to 1.675	No	ns	>0.9999
153	0.04577	-1.524 to 1.615	No	ns	>0.9999
154	-0.1927	-1.762 to 1.377	No	ns	>0.9999
155	-0.1416	-1.711 to 1.428	No	ns	>0.9999
156	-0.1053	-1.675 to 1.464	No	ns	>0.9999
157	-0.1428	-1.712 to 1.427	No	ns	>0.9999
158	0.2225	-1.347 to 1.792	No	ns	>0.9999
159	0.4210	-1.148 to 1.990	No	ns	>0.9999
160	0.2989	-1.270 to 1.868	No	ns	>0.9999
161	0.3435	-1.226 to 1.913	No	ns	>0.9999
162	0.4896	-1.080 to 2.059	No	ns	>0.9999
163	0.4112	-1.158 to 1.980	No	ns	>0.9999
164	0.1765	-1.393 to 1.746	No	ns	>0.9999
165	0.02417	-1.545 to 1.593	No	ns	>0.9999
166	0.01922	-1.550 to 1.589	No	ns	>0.9999
167	0.1414	-1.428 to 1.711	No	ns	>0.9999
168	0.1849	-1.384 to 1.754	No	ns	>0.9999
169	0.1906	-1.379 to 1.760	No	ns	>0.9999
170	0.2266	-1.343 to 1.796	No	ns	>0.9999
171	0.2555	-1.314 to 1.825	No	ns	>0.9999
172	0.2866	-1.283 to 1.856	No	ns	>0.9999
173	0.2782	-1.291 to 1.848	No	ns	>0.9999
174	0.2838	-1.286 to 1.853	No	ns	>0.9999
175	0.3901	-1.179 to 1.959	No	ns	>0.9999
176	0.5244	-1.045 to 2.094	No	ns	>0.9999
177	0.5021	-1.067 to 2.071	No	ns	>0.9999
178	0.2040	-1.365 to 1.773	No	ns	>0.9999
179	0.1525	-1.417 to 1.722	No	ns	>0.9999
180	0.07663	-1.493 to 1.646	No	ns	>0.9999
181	0.1202	-1.449 to 1.689	No	ns	>0.9999
182	-0.06235	-1.632 to 1.507	No	ns	>0.9999
183	-0.1550	-1.724 to 1.414	No	ns	>0.9999
184	0.1200	-1.449 to 1.689	No	ns	>0.9999
185	0.3601	-1.209 to 1.929	No	ns	>0.9999
186	0.2735	-1.296 to 1.843	No	ns	>0.9999
187	0.06557	-1.504 to 1.635	No	ns	>0.9999
188	-0.006367	-1.576 to 1.563	No	ns	>0.9999
189	0.1482	-1.421 to 1.717	No	ns	>0.9999
190	0.1683	-1.401 to 1.738	No	ns	>0.9999
191	0.2178	-1.351 to 1.787	No	ns	>0.9999
192	0.2063	-1.363 to 1.776	No	ns	>0.9999
193	0.1819	-1.387 to 1.751	No	ns	>0.9999
194	0.1470	-1.422 to 1.716	No	ns	>0.9999
195	0.1175	-1.452 to 1.687	No	ns	>0.9999
196	0.08248	-1.487 to 1.652	No	ns	>0.9999
197	0.05312	-1.516 to 1.622	No	ns	>0.9999
198	-0.02153	-1.591 to 1.548	No	ns	>0.9999
199	-0.02647	-1.596 to 1.543	No	ns	>0.9999

	200	-0.05638	-1.626 to 1.513	No	ns	>0.9999
	201	0.01135	-1.558 to 1.581	No	ns	>0.9999
	202	0.02795	-1.541 to 1.597	No	ns	>0.9999
	203	0.06480	-1.505 to 1.634	No	ns	>0.9999
	204	0.08737	-1.482 to 1.657	No	ns	>0.9999
	205	0.1054	-1.464 to 1.675	No	ns	>0.9999
	206	0.1315	-1.438 to 1.701	No	ns	>0.9999
	207	0.1754	-1.394 to 1.745	No	ns	>0.9999
	208	0.2154	-1.354 to 1.785	No	ns	>0.9999
	209	0.2319	-1.337 to 1.801	No	ns	>0.9999
	210	0.2664	-1.303 to 1.836	No	ns	>0.9999
	211	0.1726	-1.397 to 1.742	No	ns	>0.9999
	212	0.2060	-1.363 to 1.775	No	ns	>0.9999
	213	-0.009050	-1.578 to 1.560	No	ns	>0.9999
	214	0.1831	-1.386 to 1.752	No	ns	>0.9999
	215	0.1034	-1.466 to 1.673	No	ns	>0.9999
	216	0.09808	-1.471 to 1.667	No	ns	>0.9999
	217	0.1122	-1.457 to 1.681	No	ns	>0.9999
	218	0.07728	-1.492 to 1.647	No	ns	>0.9999
	219	0.04660	-1.523 to 1.616	No	ns	>0.9999
	220	0.04433	-1.525 to 1.614	No	ns	>0.9999
	221	0.06770	-1.502 to 1.637	No	ns	>0.9999
	222	0.1077	-1.462 to 1.677	No	ns	>0.9999
	223	0.1143	-1.455 to 1.684	No	ns	>0.9999
	224	0.1089	-1.460 to 1.678	No	ns	>0.9999
	225	0.07860	-1.491 to 1.648	No	ns	>0.9999
	226	0.04960	-1.520 to 1.619	No	ns	>0.9999
	227	0.03652	-1.533 to 1.606	No	ns	>0.9999
	228	0.04048	-1.529 to 1.610	No	ns	>0.9999
	229	0.04085	-1.528 to 1.610	No	ns	>0.9999
	230	0.04428	-1.525 to 1.614	No	ns	>0.9999
	231	0.04500	-1.524 to 1.614	No	ns	>0.9999
	232	0.01142	-1.558 to 1.581	No	ns	>0.9999
	233	-0.04212	-1.611 to 1.527	No	ns	>0.9999
	234	-0.06228	-1.632 to 1.507	No	ns	>0.9999
	235	-0.06130	-1.631 to 1.508	No	ns	>0.9999
	236	0.03678	-1.533 to 1.606	No	ns	>0.9999
	237	0.06193	-1.507 to 1.631	No	ns	>0.9999
	238	0.08870	-1.481 to 1.658	No	ns	>0.9999
	239	0.1230	-1.446 to 1.692	No	ns	>0.9999
	240	0.1599	-1.409 to 1.729	No	ns	>0.9999
	241	0.1866	-1.383 to 1.756	No	ns	>0.9999
	242	0.2026	-1.367 to 1.772	No	ns	>0.9999
	243	0.1846	-1.385 to 1.754	No	ns	>0.9999
	244	0.1943	-1.375 to 1.764	No	ns	>0.9999
	245	0.2541	-1.315 to 1.823	No	ns	>0.9999
	246	0.3629	-1.206 to 1.932	No	ns	>0.9999
	247	0.2525	-1.317 to 1.822	No	ns	>0.9999
	248	0.8441	-0.7252 to 2.413	No	ns	>0.9999
	249	1.800	0.2307 to 3.369	Yes	**	0.0051
	250	1.981	0.4121 to 3.551	Yes	***	0.0007
	251	1.504	-0.06485 to 3.074	No	ns	0.0890
	252	1.468	-0.1018 to 3.037	No	ns	0.1217
	253	1.063	-0.5060 to 2.633	No	ns	0.9534
	254	-0.1437	-1.713 to 1.426	No	ns	>0.9999
	255	-0.2500	-1.819 to 1.319	No	ns	>0.9999
	256	0.03272	-1.537 to 1.602	No	ns	>0.9999
	257	-0.3874	-1.957 to 1.182	No	ns	>0.9999
	258	-0.4291	-1.998 to 1.140	No	ns	>0.9999
	259	-0.04410	-1.613 to 1.525	No	ns	>0.9999
	260	0.5205	-1.049 to 2.090	No	ns	>0.9999
	261	0.4462	-1.123 to 2.016	No	ns	>0.9999

	262	0.2288	-1.340 to 1.798	No	ns	>0.9999
	263	0.2676	-1.302 to 1.837	No	ns	>0.9999
	264	0.1012	-1.468 to 1.670	No	ns	>0.9999
	265	0.1285	-1.441 to 1.698	No	ns	>0.9999
	266	0.1797	-1.390 to 1.749	No	ns	>0.9999
	267	0.2085	-1.361 to 1.778	No	ns	>0.9999
	268	0.1588	-1.411 to 1.728	No	ns	>0.9999
	269	0.1300	-1.439 to 1.699	No	ns	>0.9999
	270	0.09563	-1.474 to 1.665	No	ns	>0.9999
	271	0.08532	-1.484 to 1.655	No	ns	>0.9999
	272	0.05353	-1.516 to 1.623	No	ns	>0.9999
	273	0.05000	-1.519 to 1.619	No	ns	>0.9999
	274	0.06108	-1.508 to 1.630	No	ns	>0.9999
	275	0.06670	-1.503 to 1.636	No	ns	>0.9999
	276	0.07000	-1.499 to 1.639	No	ns	>0.9999
	277	0.08763	-1.482 to 1.657	No	ns	>0.9999
	278	0.09057	-1.479 to 1.660	No	ns	>0.9999
	279	0.1078	-1.461 to 1.677	No	ns	>0.9999
	280	0.1234	-1.446 to 1.693	No	ns	>0.9999
	281	0.1413	-1.428 to 1.711	No	ns	>0.9999
	282	0.1514	-1.418 to 1.721	No	ns	>0.9999
	283	0.1191	-1.450 to 1.688	No	ns	>0.9999
	284	0.07558	-1.494 to 1.645	No	ns	>0.9999
	285	0.07423	-1.495 to 1.644	No	ns	>0.9999
	286	0.07518	-1.494 to 1.644	No	ns	>0.9999
	287	0.1054	-1.464 to 1.675	No	ns	>0.9999
	288	0.1002	-1.469 to 1.670	No	ns	>0.9999
	289	0.08975	-1.480 to 1.659	No	ns	>0.9999
	290	0.07340	-1.496 to 1.643	No	ns	>0.9999
	291	0.05165	-1.518 to 1.621	No	ns	>0.9999
	292	0.04182	-1.527 to 1.611	No	ns	>0.9999
	293	0.01907	-1.550 to 1.588	No	ns	>0.9999
	294	0.004067	-1.565 to 1.573	No	ns	>0.9999
	295	-0.01487	-1.584 to 1.554	No	ns	>0.9999
	296	-0.02013	-1.589 to 1.549	No	ns	>0.9999

Table S2. Statistical analysis of RMSF plots from wt and B.1.1.7 spike protein residues 800-900 (n = 6; two-way ANOVA; significance assumed for *p<0.05).

Compare each cell mean with the other cell mean in that row					
Number of families	1				
Number of comparisons per family	100				
Alpha	0.05				
Sidak's multiple comparisons test	Mean Diff,	95,00% CI of diff,	Significant?	Summary	Adjusted P Value
resdiue number					
800	-0.1280	-1.995 to 1.739	No	ns	>0.9999
801	-0.2166	-2.084 to 1.650	No	ns	>0.9999
802	-0.2190	-2.086 to 1.648	No	ns	>0.9999
803	-0.2493	-2.116 to 1.618	No	ns	>0.9999
804	-0.3069	-2.174 to 1.560	No	ns	>0.9999
805	-0.2610	-2.128 to 1.606	No	ns	>0.9999
806	-0.2261	-2.093 to 1.641	No	ns	>0.9999
807	-0.1018	-1.969 to 1.765	No	ns	>0.9999
808	0.06743	-1.799 to 1.934	No	ns	>0.9999
809	-0.04395	-1.911 to 1.823	No	ns	>0.9999
810	-0.1864	-2.053 to 1.681	No	ns	>0.9999
811	-0.2875	-2.154 to 1.579	No	ns	>0.9999
812	-0.3187	-2.186 to 1.548	No	ns	>0.9999
813	-0.4475	-2.314 to 1.419	No	ns	>0.9999
814	-0.3771	-2.244 to 1.490	No	ns	>0.9999
815	-0.3110	-2.178 to 1.556	No	ns	>0.9999
816	-0.2772	-2.144 to 1.590	No	ns	>0.9999
817	-0.2562	-2.123 to 1.611	No	ns	>0.9999
818	-0.2571	-2.124 to 1.610	No	ns	>0.9999
819	-0.2522	-2.119 to 1.615	No	ns	>0.9999
820	-0.2137	-2.081 to 1.653	No	ns	>0.9999
821	-0.2046	-2.071 to 1.662	No	ns	>0.9999
822	-0.1648	-2.032 to 1.702	No	ns	>0.9999
823	-0.2029	-2.070 to 1.664	No	ns	>0.9999
824	-0.2830	-2.150 to 1.584	No	ns	>0.9999
825	-0.3213	-2.188 to 1.546	No	ns	>0.9999
826	-0.1972	-2.064 to 1.670	No	ns	>0.9999
827	-0.3541	-2.221 to 1.513	No	ns	>0.9999
828	-0.4093	-2.276 to 1.458	No	ns	>0.9999
829	-0.9103	-2.777 to 0.9567	No	ns	>0.9999
830	-1.089	-2.956 to 0.7779	No	ns	0.9862
831	-1.212	-3.079 to 0.6549	No	ns	0.9084
832	-1.656	-3.522 to 0.2113	No	ns	0.1845
833	-1.782	-3.649 to 0.08476	No	ns	0.0867
834	-1.768	-3.635 to 0.09891	No	ns	0.0947
835	-2.051	-3.918 to -0.1844	Yes	*	0.0137
836	-2.291	-4.158 to -0.4240	Yes	**	0.0021
837	-2.393	-4.260 to -0.5262	Yes	***	0.0009
838	-2.537	-4.404 to -0.6704	Yes	***	0.0003
839	-1.878	-3.745 to -0.01122	Yes	*	0.0464
840	-2.151	-4.017 to -0.2836	Yes	**	0.0065
841	-2.035	-3.902 to -0.1686	Yes	*	0.0154
842	-2.393	-4.260 to -0.5264	Yes	***	0.0009
843	-2.179	-4.046 to -0.3123	Yes	**	0.0052
844	-1.754	-3.620 to 0.1133	No	ns	0.1036
845	-1.753	-3.620 to 0.1140	No	ns	0.1040
846	-1.470	-3.337 to 0.3967	No	ns	0.4579
847	-1.228	-3.095 to 0.6391	No	ns	0.8905
848	-1.048	-2.915 to 0.8192	No	ns	0.9942
849	-0.9947	-2.862 to 0.8722	No	ns	0.9985

850	-0.7876	-2.654 to 1.079	No	ns	>0.9999
851	-1.005	-2.872 to 0.8620	No	ns	0.9980
852	-0.8561	-2.723 to 1.011	No	ns	>0.9999
853	-0.5983	-2.465 to 1.269	No	ns	>0.9999
854	-0.3093	-2.176 to 1.558	No	ns	>0.9999
855	-0.04725	-1.914 to 1.820	No	ns	>0.9999
856	-0.07502	-1.942 to 1.792	No	ns	>0.9999
857	-0.1309	-1.998 to 1.736	No	ns	>0.9999
858	-0.1545	-2.021 to 1.712	No	ns	>0.9999
859	-0.2139	-2.081 to 1.653	No	ns	>0.9999
860	-0.2246	-2.091 to 1.642	No	ns	>0.9999
861	-0.2097	-2.077 to 1.657	No	ns	>0.9999
862	-0.2924	-2.159 to 1.575	No	ns	>0.9999
863	-0.3368	-2.204 to 1.530	No	ns	>0.9999
864	-0.3380	-2.205 to 1.529	No	ns	>0.9999
865	-0.3322	-2.199 to 1.535	No	ns	>0.9999
866	-0.2914	-2.158 to 1.576	No	ns	>0.9999
867	-0.3157	-2.183 to 1.551	No	ns	>0.9999
868	-0.3822	-2.249 to 1.485	No	ns	>0.9999
869	-0.3861	-2.253 to 1.481	No	ns	>0.9999
870	-0.3807	-2.248 to 1.486	No	ns	>0.9999
871	-0.3803	-2.247 to 1.487	No	ns	>0.9999
872	-0.3900	-2.257 to 1.477	No	ns	>0.9999
873	-0.4014	-2.268 to 1.466	No	ns	>0.9999
874	-0.3739	-2.241 to 1.493	No	ns	>0.9999
875	-0.3471	-2.214 to 1.520	No	ns	>0.9999
876	-0.3396	-2.206 to 1.527	No	ns	>0.9999
877	-0.3380	-2.205 to 1.529	No	ns	>0.9999
878	-0.3264	-2.193 to 1.540	No	ns	>0.9999
879	-0.2904	-2.157 to 1.576	No	ns	>0.9999
880	-0.2508	-2.118 to 1.616	No	ns	>0.9999
881	-0.2283	-2.095 to 1.639	No	ns	>0.9999
882	-0.1984	-2.065 to 1.669	No	ns	>0.9999
883	-0.1742	-2.041 to 1.693	No	ns	>0.9999
884	-0.1965	-2.063 to 1.670	No	ns	>0.9999
885	-0.08338	-1.950 to 1.784	No	ns	>0.9999
886	-0.2021	-2.069 to 1.665	No	ns	>0.9999
887	-0.3500	-2.217 to 1.517	No	ns	>0.9999
888	-0.4662	-2.333 to 1.401	No	ns	>0.9999
889	-0.5197	-2.387 to 1.347	No	ns	>0.9999
890	-0.3981	-2.265 to 1.469	No	ns	>0.9999
891	-0.3144	-2.181 to 1.552	No	ns	>0.9999
892	-0.1745	-2.041 to 1.692	No	ns	>0.9999
893	-0.1717	-2.039 to 1.695	No	ns	>0.9999
894	-0.1517	-2.019 to 1.715	No	ns	>0.9999
895	-0.1323	-1.999 to 1.735	No	ns	>0.9999
896	-0.1209	-1.988 to 1.746	No	ns	>0.9999
897	-0.1356	-2.002 to 1.731	No	ns	>0.9999
898	-0.1097	-1.977 to 1.757	No	ns	>0.9999
899	-0.09742	-1.964 to 1.769	No	ns	>0.9999

Table S3. Statistical analysis of contacts between residues from the receptor binding domain and ACE2 from wt and B.1.1.7 (n = 4; two-way ANOVA; significance assumed for *p<0.05).

Compare each cell mean with the other cell mean in that row					
Number of families	1				
Number of comparisons per family	39				
Alpha	0.05				
Sidak's multiple comparisons test	Mean Diff,	95,00% CI of diff,	Significant?	Summary	Adjusted P Value
wt - B.1.1.7					
Gln24-Ala475	-3.966	-36.71 to 28.77	No	ns	>0.9999
Gln24-Gly476	-6.900	-60.96 to 47.16	No	ns	>0.9999
Gln24-Phe486	-4.431	-56.96 to 48.10	No	ns	>0.9999
Gln24-Asn487	-3.633	-39.32 to 32.06	No	ns	>0.9999
Gln24-Tyr489	1.424	-27.42 to 30.27	No	ns	>0.9999
Thr27-Phe456	-27.09	-227.5 to 173.3	No	ns	>0.9999
Thr27-Tyr473	-1.580	-11.80 to 8.639	No	ns	>0.9999
Thr27-Ala475	-0.2872	-16.29 to 15.71	No	ns	>0.9999
Thr27-Tyr489	-0.2933	-39.37 to 38.78	No	ns	>0.9999
Asp30-Lys417	-2.107	-40.08 to 35.86	No	ns	>0.9999
Asp30-Leu455	1.832	-37.21 to 40.87	No	ns	>0.9999
Asp30-Phe456	-1.802	-4.475 to 0.8716	No	ns	0.2915
Lys31-Leu455	3.215	-27.90 to 34.33	No	ns	>0.9999
Lys31-Phe456	-0.2198	-13.34 to 12.90	No	ns	>0.9999
Lys31-Tyr489	-1.140	-42.64 to 40.36	No	ns	>0.9999
Lys31-Phe490	0.2721	-34.14 to 34.69	No	ns	>0.9999
Lys31-Gln493	5.029	-20.40 to 30.46	No	ns	>0.9999
His34-Tyr453	7.590	-23.02 to 38.20	No	ns	0.9928
His34-Leu455	-1.827	-86.36 to 82.71	No	ns	>0.9999
His34-Gln493	-4.108	-30.22 to 22.00	No	ns	>0.9999
His34-Tyr495	6.822	-47.09 to 60.73	No	ns	>0.9999
Glu35-Gln493	3.989	-13.29 to 21.27	No	ns	>0.9999
Glu37-Tyr505	6.696	-12.10 to 25.49	No	ns	0.7336
Asp38-Tyr449	20.14	4.753 to 35.52	Yes	*	0.0201
Asp38-Gln498	16.06	-8.407 to 40.52	No	ns	0.1612
Tyr41-Gln498	24.73	3.697 to 45.76	Yes	*	0.0260
Tyr41-Thr500	5.704	-5.204 to 16.61	No	ns	0.6281
Tyr41-Asn/Tyr501	-1.294	-19.15 to 16.56	No	ns	>0.9999
Leu79-Val483	4.129	-27.98 to 36.24	No	ns	>0.9999
Leu79-Gly485	3.031	-22.75 to 28.81	No	ns	>0.9999
Leu79-Phe486	-1.775	-19.09 to 15.54	No	ns	>0.9999
Lys353-Gly496	15.10	8.848 to 21.35	Yes	***	0.0004
Lys353-Phe497	15.54	2.414 to 28.68	Yes	*	0.0307
Lys353-Gln498	43.86	41.70 to 46.03	Yes	****	<0.0001
Lys353-Asn/Tyr501	-31.08	-36.95 to -25.21	Yes	****	<0.0001
Lys353-Gly502	0.1488	-1.268 to 1.566	No	ns	>0.9999
Lys353-Tyr505	5.197	-14.64 to 25.03	No	ns	0.9954
Gly354-Tyr505	5.532	-31.56 to 42.62	No	ns	0.9998
Ala387-Tyr505	4.640	-44.51 to 53.80	No	ns	>0.9999

Table S4. Statistical analysis of electrostatic interaction energy of residues from the receptor binding domain comparing wt and B.1.1.7 (n = 4; two-way ANOVA; significance assumed for *p<0.05).

Compare each cell mean with the other cell mean in that row					
Number of families	1				
Number of comparisons per family	18				
Alpha	0.05				
Sidak's multiple comparisons test	Mean Diff,	95,00% CI of diff,	Significant?	Summary	Adjusted P Value
electrostatic wt - electrostatic B.1.1.7					
Lys417	2.002	-7.629 to 11.63	No	ns	>0.9999
Tyr449	-9.196	-18.83 to 0.4345	No	ns	0.0737
Tyr453	-0.8195	-10.45 to 8.811	No	ns	>0.9999
Leu455	-0.04517	-9.676 to 9.585	No	ns	>0.9999
Phe456	0.2792	-9.351 to 9.909	No	ns	>0.9999
Tyr473	0.2544	-9.376 to 9.885	No	ns	>0.9999
Ala475	0.6749	-8.955 to 10.31	No	ns	>0.9999
Gly476	0.7864	-8.844 to 10.42	No	ns	>0.9999
Phe486	0.01221	-9.618 to 9.643	No	ns	>0.9999
Asn487	0.3207	-9.310 to 9.951	No	ns	>0.9999
Tyr489	0.06032	-9.570 to 9.691	No	ns	>0.9999
Gln493	4.088	-5.543 to 13.72	No	ns	0.9775
Gly496	-8.394	-18.02 to 1.236	No	ns	0.1446
Gln498	-18.40	-28.03 to -8.771	Yes	****	<0.0001
Thr500	0.4097	-9.221 to 10.04	No	ns	>0.9999
Asn/Tyr501	-6.797	-16.43 to 2.833	No	ns	0.4389
Gly502	-2.077	-11.71 to 7.553	No	ns	>0.9999
Tyr505	-1.129	-10.76 to 8.502	No	ns	>0.9999

Table S5. Statistical analysis of electrostatic interaction energy of residues from ACE2 comparing wt and B.1.1.7 (n = 4; two-way ANOVA; significance assumed for *p<0.05).

Compare each cell mean with the other cell mean in that row					
Number of families	1				
Number of comparisons per family	17				
Alpha	0.05				
Sidak's multiple comparisons test	Mean Diff,	95,00% CI of diff,	Significant?	Summary	Adjusted P Value
electrostatic wt - electrostatic B.1.1.7					
Gln24	1.291	-8.879 to 11.46	No	ns	>0.9999
Thr27	0.08892	-10.08 to 10.26	No	ns	>0.9999
Phe28	0.09204	-10.08 to 10.26	No	ns	>0.9999
Asp30	4.740	-5.430 to 14.91	No	ns	0.9401
Lys31	0.3279	-9.841 to 10.50	No	ns	>0.9999
His34	-4.305	-14.47 to 5.864	No	ns	0.9740
Glu35	-1.158	-11.33 to 9.011	No	ns	>0.9999
Asp38	-8.984	-19.15 to 1.185	No	ns	0.1303
Tyr41	-1.274	-11.44 to 8.895	No	ns	>0.9999
Gln42	-1.510	-11.68 to 8.659	No	ns	>0.9999
Met82	0.008097	-10.16 to 10.18	No	ns	>0.9999
Tyr83	-0.6152	-10.78 to 9.554	No	ns	>0.9999
Asn330	0.7600	-9.409 to 10.93	No	ns	>0.9999
Lys353	-28.08	-38.25 to -17.91	Yes	****	<0.0001
Gly354	-0.5079	-10.68 to 9.661	No	ns	>0.9999
Asp355	-0.8822	-11.05 to 9.287	No	ns	>0.9999
Arg357	0.2824	-9.887 to 10.45	No	ns	>0.9999

Table S6. Statistical analysis of van der Waals interaction energy of residues from the receptor binding domain comparing wt and B.1.1.7 (n = 4; two-way ANOVA; significance assumed for *p<0.05).

Compare each cell mean with the other cell mean in that row					
Number of families	1				
Number of comparisons per family	18				
Alpha	0.05				
Sidak's multiple comparisons test	Mean Diff,	95,00% CI of diff,	Significant?	Summary	Adjusted P Value
VdW wt - VdW B.1.1.7					
Lys417	-0.1159	-0.8238 to 0.5920	No	ns	0.9996
Tyr449	-0.5646	-1.674 to 0.5452	No	ns	0.5767
Tyr453	-0.1531	-0.6207 to 0.3144	No	ns	0.9561
Leu455	-0.1551	-0.9608 to 0.6506	No	ns	0.9995
Phe456	0.2132	-0.4923 to 0.9186	No	ns	0.8694
Tyr473	0.1292	-0.9458 to 1.204	No	ns	>0.9999
Ala475	0.2620	-2.877 to 3.401	No	ns	>0.9999
Gly476	0.4282	-4.984 to 5.840	No	ns	>0.9999
Phe486	-0.04765	-0.6225 to 0.5272	No	ns	>0.9999
Asn487	0.1392	-0.6292 to 0.9076	No	ns	0.9978
Tyr489	-0.02050	-0.9177 to 0.8767	No	ns	>0.9999
Gln493	-0.2753	-1.040 to 0.4894	No	ns	0.8832
Gly496	-0.2742	-0.7783 to 0.2299	No	ns	0.4956
Gln498	-1.555	-2.501 to -0.6099	Yes	**	0.0039
Thr500	-0.1012	-0.8328 to 0.6305	No	ns	>0.9999
Asn/Tyr501	1.917	1.276 to 2.559	Yes	***	0.0001
Gly502	0.1328	-0.2838 to 0.5493	No	ns	0.7502
Tyr505	-0.8373	-3.986 to 2.311	No	ns	0.9887

Table S7. Statistical analysis of electrostatic interaction energy of residues from ACE2 comparing wt and B.1.1.7 (n = 4; two-way ANOVA; significance assumed for *p<0.05).

Compare each cell mean with the other cell mean in that row					
Number of families	1				
Number of comparisons per family	17				
Alpha	0.05				
Sidak's multiple comparisons test	Mean Diff,	95,00% CI of diff,	Significant?	Summary	Adjusted P Value
electrostatic wt - electrostatic B.1.1.7					
Gln24	0.3199	-2.765 to 3.405	No	ns	>0.9999
Thr27	0.2025	-0.8693 to 1.274	No	ns	0.9924
Phe28	-0.08288	-1.123 to 0.9569	No	ns	>0.9999
Asp30	-0.1104	-0.4978 to 0.2770	No	ns	0.9745
Lys31	-0.04280	-1.378 to 1.292	No	ns	>0.9999
His34	-0.5685	-1.175 to 0.03814	No	ns	0.0651
Glu35	-0.5276	-1.109 to 0.05356	No	ns	0.0772
Asp38	-0.7532	-2.592 to 1.086	No	ns	0.6362
Tyr41	-1.162	-2.035 to -0.2886	Yes	*	0.0191
Gln42	-0.9193	-1.665 to -0.1739	Yes	*	0.0184
Met82	-0.1952	-1.267 to 0.8763	No	ns	0.9934
Tyr83	-0.07057	-0.8227 to 0.6816	No	ns	>0.9999
Asn330	0.1921	-0.4348 to 0.8190	No	ns	0.9457
Lys353	0.4709	-2.538 to 3.480	No	ns	0.9951
Gly354	0.007700	-0.6917 to 0.7071	No	ns	>0.9999
Asp355	0.1154	-0.3833 to 0.6140	No	ns	0.9726
Arg357	0.1624	-0.1969 to 0.5216	No	ns	0.6094
